# Association between body mass index and prognosis of patients hospitalized with heart failure

**DOI:** 10.1038/s41598-020-73640-w

**Published:** 2020-10-07

**Authors:** Yuta Seko, Takao Kato, Takeshi Morimoto, Hidenori Yaku, Yasutaka Inuzuka, Yodo Tamaki, Neiko Ozasa, Masayuki Shiba, Erika Yamamoto, Yusuke Yoshikawa, Yugo Yamashita, Takeshi Kitai, Ryoji Taniguchi, Moritake Iguchi, Kazuya Nagao, Takafumi Kawai, Akihiro Komasa, Ryusuke Nishikawa, Yuichi Kawase, Takashi Morinaga, Mamoru Toyofuku, Yutaka Furukawa, Kenji Ando, Kazushige Kadota, Yukihito Sato, Koichiro Kuwahara, Takeshi Kimura

**Affiliations:** 1 Department of Cardiovascular Medicine, Kyoto University Graduate School of Medicine, 54 Shogoin Kawahara-cho, Sakyo-ku, Kyoto, 606-8507 Japan; 2 Clinical Epidemiology, Hyogo College of Medicine, Nishinomiya, Japan; 3 Cardiovascular Medicine, Shiga General Hospital, Moriyama, Japan; 4 Division of Cardiology, Tenri Hospital, Nara, Japan; 5 Department of Cardiovascular Medicine, Kobe City Medical Center General Hospital, Hyogo, Japan; 6 Department of Cardiology, Hyogo Prefectural Amagasaki General Medical Center, Hyogo, Japan; 7 Department of Cardiology, National Hospital Organization Kyoto Medical Center, Kyoto, Japan; 8 Department of Cardiology, Osaka Red Cross Hospital, Osaka, Japan; 9 Department of Cardiology, Kishiwada City Hospital, Osaka, Japan; 10 Department of Cardiology, Kansai Electric Power Hospital, Osaka, Japan; 11 Department of Cardiology, Shizuoka General Hospital, Shizuoka, Japan; 12 Department of Cardiology, Kurashiki Central Hospital, Okayama, Japan; 13 Department of Cardiology, Kokura Memorial Hospital, Fukuoka, Japan; 14 Department of Cardiology, Japanese Red Cross Wakayama Medical Center, Wakayama, Japan; 15 Department of Cardiovascular Medicine, Shinshu University Graduate School of Medicine, Nagano, Japan

**Keywords:** Cardiovascular diseases, Heart failure, Cardiology

## Abstract

The prognostic implications of very low body mass index (BMI) values remain unclear in patients with acute decompensated heart failure (ADHF). This study aimed to investigate the prognostic impact of BMI classification based on the World Health Organization criteria in patients with ADHF. Among 3509 patients with ADHF and available BMI data at discharge in 19 participating hospitals in Japan between October 2014 and March 2016, the study population was divided into five groups; (1) Severely underweight: BMI < 16 kg/m^2^, (2) Underweight: BMI ≥ 16 kg/m^2^ and < 18.5 kg/m^2^, (3) Normal weight: BMI ≥ 18.5 kg/m^2^ and < 25 kg/m^2^, (4) Overweight: BMI ≥ 25 kg/m^2^ and < 30 kg/m^2^ (5) Obese: BMI ≥ 30 kg/m^2^. The primary outcome measure was all-cause death. The median follow-up duration was 471 days, with 96.4% follow up at 1-year. The cumulative 1-year incidence of all-cause death was higher in underweight groups, and lower in overweight groups (Severely underweight: 36.3%, Underweight: 23.9%, Normal weight: 14.4%, Overweight: 7.9%, and Obese: 9.0%, *P* < 0.001). After adjusting confounders, the excess mortality risk remained significant in the severely underweight group (HR, 2.32; 95%CI, 1.83–2.94; *P* < 0.001), and in the underweight group (HR, 1.31; 95%CI, 1.08–1.59; *P* = 0.005) relative to the normal weight group, while the lower mortality risk was no longer significant in the overweight group (HR, 0.82; 95%CI, 0.62–1.10; *P* = 0.18) and in the obese group (HR, 1.09; 95%CI, 0.65–1.85; *P* = 0.74). Very low BMI was associated with a higher risk for one-year mortality after discharge in patients with ADHF.

## Introduction

Obesity, or a higher body mass index (BMI) is associated with an increased risk of death and cardiovascular events including heart failure (HF) in the general population^1–3^. On the other hand, a higher BMI has been demonstrated to have a paradoxical association with a decreased risk of mortality in patients with HF^4–8^. Previous studies have also demonstrated higher mortality rates in HF patients with a lower BMI^4–8^. There is a global trend for progressive aging of HF patients^9,10^. BMI values are much lower in elderly patients than in younger individuals, and the overlap of aging and low BMI has been most prominently seen in Japan^11^. Although the association with low BMI and mortality in patients with HF has been confirmed, there is a paucity of data on the association between a severely low BMI at discharge and mortality in both chronic and acute decompensated HF (ADHF) patients across the world. Identifying the characteristics and prognosis of patients with a severely low BMI may be useful for the improvement of the management of HF, especially in elderly patients. In addition, the association between obesity and mortality or HF hospitalization is uncertain in the patients with HF in Japan because of the very small number of obese patients in Japan. Thus, we aimed to examine the association between the BMI status at discharge based on the World Health Organization (WHO) standard and the 1-year mortality or HF hospitalization, along with the cardiovascular and non-cardiovascular death, using a large contemporary all-comer registry of patients with ADHF hospitalization in Japan.

## Method

### Study design, setting, and population

The Kyoto Congestive Heart Failure (KCHF) registry is a physician-initiated, prospective, observational, multicenter cohort study that enrolled consecutive patients who were hospitalized for ADHF for the first time between 1 October 2014 and 31 March 2016 without any exclusion criteria. These patients were admitted into 19 secondary and tertiary hospitals, including rural and urban, large and small institutions, throughout Japan. The overall design of the KCHF study and patient enrolment has been previously described in detail^11–16^.

We enrolled consecutive patients with ADHF as defined by the modified Framingham criteria admitted to the participating centers, who underwent heart failure-specific treatment involving intravenous drugs within 24 h of hospital presentation. Patient records were anonymized before analysis. Data analysis was conducted from February 2020 to March 2020.

**Figure 1:**
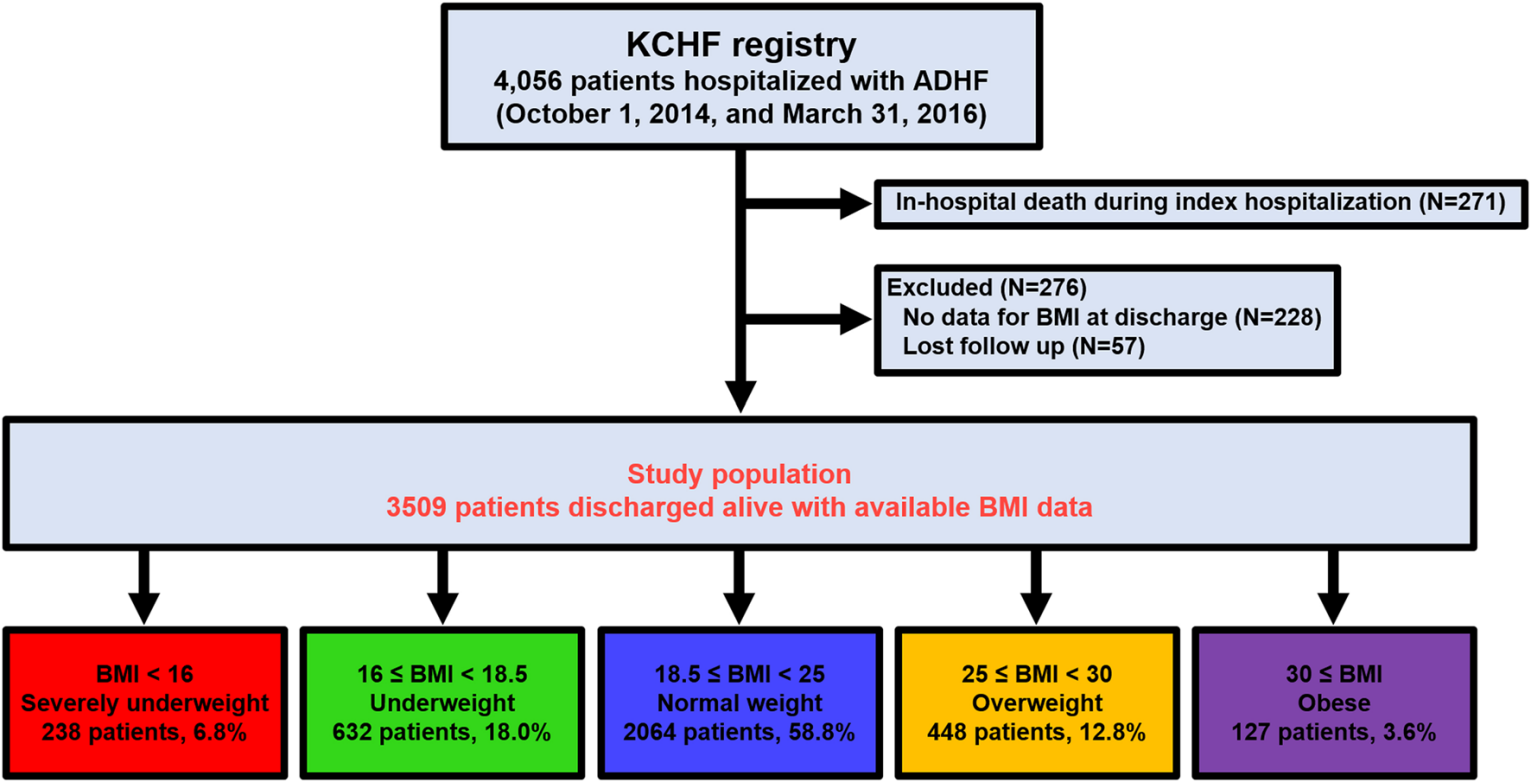
Study flowchart. *ADHF* acute decompensated heart failure, *BMI* body mass index, *KCHF* Kyoto Congestive Heart Failure.

Among 4056 patients enrolled in the KCHF registry, the current study population consisted of 3509 patients who were discharged alive and whose BMI was calculated at discharge, excluding 271 patients who died during the index hospitalization, 228 patients whose BMI at discharge was not available (Supplementary Table 1), and 57 patients were excluded because of missing follow-up data after discharge. (Fig. 1). We stratified the patients into 5 groups according to BMI at discharge based on the WHO standard^17^; (1) Severely underweight: BMI < 16 kg/m^2^, (2) Underweight: BMI ≥ 16 kg/m^2^ and < 18.5 kg/m^2^, (3) Normal weight: BMI ≥ 18.5 kg/m^2^ and < 25 kg/m^2^, (4) Overweight: BMI ≥ 25 kg/m^2^ and < 30 kg/m^2^, and (5) Obese: BMI ≥ 30 kg/m^2^.

### Definitions

We collected data on patient demographics, medical history, underlying heart disease, pre-hospital activities, socioeconomic status, signs, symptoms, medications, laboratory tests at hospital presentation, electrocardiogram, echocardiography during hospitalization^11,13,14^.

The detailed definitions of baseline patient characteristics were as follows: BMI was calculated as weight in kilograms divided by the square of the height in meters^17^. Anemia was defined using the WHO criteria (hemoglobin < 12.0 g/dL in women and < 13.0 g/dL in men). Chronic kidney disease was defined as estimated glomerular filtration rate (eGFR) < 60 mL/min/1.73 m^2^ at admission. Renal dysfunction was defined as estimated glomerular filtration rate (eGFR) < 30 mL/min/1.73 m^2^ based on the chronic kidney disease grades^11^. HF was classified according to left ventricular ejection fraction (LVEF), as HF with preserved LVEF (LVEF ≥ 50%), HF with mid-range LVEF (40% ≤ LVEF < 50%), and HF with reduced LVEF (LVEF < 40%)^18^.

### Outcomes

One-year clinical follow-up data with an allowance of one month, were collected in October 2017. The attending physicians or research assistants at each participating hospital collected clinical events after the index of hospitalization from hospital charts or by contacting patients, their relatives or their referring physicians with consent.

The primary outcome measure for the present analysis was all-cause death after discharge from the index hospitalization. The secondary outcome measures included cardiovascular death, non-cardiovascular death, and HF hospitalization after discharge from the index hospitalization. The causes of death were classified according to the VARC (Valve Academic Research Consortium) definitions^19^ and were adjudicated by a clinical event committee^11–14^.

### Statistical analysis

We evaluated BMI as a categorical variable (severely underweight, underweight, normal weight [reference], overweight, and obese).

The categorical variables are presented as numbers and percentages. The continuous variables are expressed as mean and standard deviation (SD) or median with interquartile range (IQR). Comparisons among 5 groups were performed using a 1-way ANOVA or Kruskal–Wallis test for continuous variables and the chi-square test for categorical variables. We regarded the date of discharge as time zero for clinical follow-up. We compared the baseline characteristics and clinical outcomes on the basis of BMI status at discharge from the index hospitalization.

The cumulative incidences of the clinical events during 1-year after discharge were estimated using the Kaplan–Meier method with the intergroup differences assessed by the log-rank test. To estimate the risk of each BMI group with normal weight group as the reference, a multivariable Cox proportional hazards model was developed for the primary and secondary outcome measures adjusting for the confounders. We included the following 24 clinically relevant risk-adjusting variables into the model: age as a continuous variable, sex, LVEF < 40% by echocardiography, variables related to medical history (etiology of HF hospitalization associated with acute coronary syndrome, previous HF hospitalization, atrial fibrillation or flutter, hypertension, diabetes mellitus, previous myocardial infarction, previous stroke, current smoking, chronic lung disease and malignant neoplasm), variables related to comorbidities (living alone, ambulatory, systolic blood pressure < 90 mmHg, heart rate < 60 bpm, eGFR < 30 ml/min/1.73m^2^, albumin < 3.0 g/dL, sodium < 135 mEq/L, and anemia), and medications at discharge (angiotensin converting enzyme inhibitors [ACEIs] or angiotensin II receptor blockers [ARBs], β-blockers, and tolvaptan), consistent with our previous reports^13,14,16^. The continuous variables were dichotomized by clinically meaningful reference values or median values. The results were expressed as a hazard ratio (HR) and 95% confidence intervals (CIs). In the post hoc subgroup analysis we evaluated the interaction between the 6 subgroup factors (age ≥ 80 years, sex, diabetes mellitus, eGFR < 30 ml/min/1.73 m^2^, LVEF < 40%, and residual edema at discharge) and the effect of BMI classification on the primary outcome measure. In the sensitivity and additional analyses, we used several classifications regarding BMI. The detailed methods were described in Supplementary methods. All statistical analyses were Y.S. and T.K. by 2 physicians (Y.S. and T.K.) and a statistician (T.M.) using JMP 14. All the reported *P* values were two tailed, and the level of statistical significance was set at *P* < 0.05.

### Ethics

The investigation conformed with the principles outlined in the Declaration of Helsinki. The study protocol was approved by the ethical committees of the Kyoto University Hospital (local identifier: E2311) and each participating hospital. A waiver of written informed consent from each patient was granted by the institutional review boards of Kyoto University and each participating center as the study met the conditions of the Japanese ethical guidelines for epidemiological study^20,21^.

## Results

### Baseline characteristics

The mean and median BMI value at discharge were 21.4 ± 4.2 kg/m^2^ and 20.9 (IQR: 18.5–23.6) kg/m^2^, respectively, and ranged from 10.5 to 55.9 kg/m^2^ (Supplementary Fig. 1). We categorized the patients into 5 groups according to BMI at discharge: Severely underweight: N = 238 (6.8%), Underweight: N = 632 (18.0%), Normal weight: N = 2064 (58.8%), Overweight: N = 448 (12.8%), and Obese: N = 127 (3.6%) (Fig. 1). Patients with lower BMI values were older and more often women, and were more likely to have malignant neoplasm, dementia, higher BNP levels, hypoalbuminemia, and anemia (Table 1). On the other hand, patients with higher BMI values were more likely to have hypertension, diabetes mellitus, dyslipidemia, previous myocardial infarction, and current smoking, and were more likely to be ambulatory (Table 1). Patients with higher BMI values more often receive angiotensin converting enzyme inhibitors (ACE-I) or angiotensin II receptor blockers (ARB) and β-blocker at discharge than those with lower BMI values.

**Table 1:** Baseline characteristics of the study subjects and transthoracic echocardiography results of the patients.

Variables	Total (N = 3509)	Severely underweight (N = 238)	Underweight (N = 632)	Normal weight (N = 2064)	Overweight (N = 448)	Obese (N = 127)	*P* value	Total N
**Clinical characteristic**								
Age*, years	77.2 ± 12.0	83.2 ± 9.5	79.9 ± 10.7	77.6 ± 11.2	72.5 ± 13.0	64.2 ± 16.9	< 0.001	3509
Age ≥ 80 years	1770 (50.4)	179 (75.2)	374 (59.2)	1035 (50.2)	154 (34.4)	28 (22.1)	< 0.001	3509
Women*	1538 (43.8)	155 (65.1)	333 (52.7)	825 (40.0)	167 (37.3)	58 (45.7)	< 0.001	3509
Body weight, kg at admission	56.7 ± 14.6	39.6 ± 6.9	45.9 ± 7.8	56.7 ± 10.0	70.8 ± 11.9	91.0 ± 20.0	< 0.001	3481
BMI at admission	22.9 ± 4.5	16.6 ± 1.9	19.1 ± 1.8	22.9 ± 2.3	28.2 ± 2.3	35.7 ± 4.9	< 0.001	3481
Body weight, kg at discharge	52.8 ± 13.5	35.3 ± 4.8	41.8 ± 5.7	53.1 ± 8.6	67.2 ± 10.0	85.7 ± 17.5	< 0.001	3509
BMI at discharge	21.4 ± 4.2	14.9 ± 0.9	17.4 ± 0.7	21.4 ± 1.8	26.7 ± 1.3	33.7 ± 4.0	< 0.001	3509
**Etiology**							< 0.001	3509
Ischemic	1147 (32.7)	54 (22.7)	178 (28.2)	698 (33.8)	182 (40.6)	35 (27.6)		
Associated with ACS*	194 (5.5)	6 (2.5)	22 (3.5)	124 (6.0)	36 (8.0)	6 (4.7)		
Not associated with ACS	953 (27.2)	48 (20.2)	156 (24.7)	574 (27.8)	146 (32.6)	29 (22.8)		
Hypertensive	870 (24.8)	58 (24.4)	123 (19.5)	518 (25.1)	129 (28.8)	42 (33.1)		
Valvular heart disease	683 (19.5)	71 (29.8)	173 (27.4)	370 (17.9)	53 (11.8)	16 (12.6)		
Cardiomyopathy	534 (15.2)	33 (13.9)	103 (16.3)	314 (15.2)	62 (13.8)	22 (17.3)		
Dilated cardiomyopathy	386 (11.0)	23 (9.7)	69 (10.9)	226 (11.0)	50 (11.2)	18 (14.2)		
Arrhythmia-related	164 (4.7)	12 (5.0)	32 (5.1)	103 (5.0)	13 (2.9)	4 (3.2)		
**Medical history**								
Heart failure hospitalization*	1272 (36.8)	101 (42.6)	247 (39.5)	714 (35.2)	160 (36.3)	50 (40.0)	0.09	3456
Hypertension*	2551 (72.7)	151 (63.5)	411 (65.0)	1538 (74.5)	358 (79.9)	93 (73.2)	< 0.001	3509
Diabetes*	1327 (37.8)	38 (16.0)	155 (24.5)	809 (39.2)	245 (54.7)	80 (63.0)	< 0.001	3509
Dyslipidemia	1393 (39.7)	65 (27.3)	197 (31.2)	827 (40.1)	233 (52.0)	71 (55.9)	< 0.001	3509
Atrial fibrillation or flutter*	1477 (42.1)	104 (43.7)	272 (43.0)	867 (42.0)	189 (42.2)	45 (35.4)	0.59	3509
VT/VF	147 (4.2)	6 (2.5)	29 (4.6)	84 (4.1)	21 (4.7)	7 (5.5)	0.59	3509
Previous myocardial infarction*	806 (23.0)	38 (16.0)	134 (21.2)	486 (23.6)	123 (27.5)	25 (19.7)	0.007	3509
Prior PCI or CABG	924 (26.3)	42 (17.7)	141 (22.3)	555 (26.9)	153 (34.2)	33 (26.0)	< 0.001	3509
Previous stroke*	548 (15.6)	32 (13.5)	106 (16.8)	331 (16.0)	68 (15.2)	11 (8.7)	0.17	3509
Current smoking*	442 (12.8)	25 (10.6)	63 (10.2)	255 (12.6)	76 (17.2)	23 (18.1)	0.003	3450
Chronic lung disease*	463 (13.2)	34 (14.3)	93 (14.7)	266 (12.9)	52 (11.6)	18 (14.2)	0.59	3509
COPD	289 (8.2)	27 (11.3)	70 (11.1)	163 (7.9)	24 (5.4)	5 (3.9)	0.001	3509
Liver cirrhosis	46 (1.3)	0 (0)	3 (0.5)	36 (1.7)	5 (1.1)	2 (1.6)	0.04	3509
Malignancy *	507 (14.4)	45 (18.9)	109 (17.3)	288 (14.0)	57 (12.7)	8(6.3)	0.003	3509
Dementia	569 (16.2)	69 (29.0)	131 (20.7)	324 (15.7)	37 (8.3)	8 (6.3)	< 0.001	3509
**Social background on admission**								
Poor medical adherence	586 (16.7)	35 (14.7)	113 (17.9)	334 (16.2)	84 (18.8)	20 (15.8)	0.53	3509
Living alone*	755 (21.5)	50 (21.0)	140 (22.2)	422 (20.5)	112 (25.0)	31 (24.4)	0.25	3509
Employed	484 (13.8)	11 (4.6)	60 (9.5)	269 (13.0)	102 (22.8)	42 (33.1)	< 0.001	3509
Public financial assistance	207 (5.9)	13 (5.5)	39 (6.2)	116 (5.6)	31 (6.9)	8 (6.3)	0.86	3509
**Daily life activities**							< 0.001	3475
Ambulatory*	2837 (81.6)	161 (68.2)	475 (76.2)	1709 (83.7)	381 (85.4)	111 (87.4)		
Use of wheelchair (outdoor only)	251 (7.2)	16 (6.8)	53 (8.5)	147 (7.2)	28 (6.3)	7 (5.5)		
Use of wheelchair (outdoor and indoor)	297 (8.5)	45 (19.1)	72 (11.6)	148 (7.2)	29 (6.5)	3 (2.4)		
Bedridden	90 (2.6)	14 (5.9)	23 (3.7)	39 (1.9)	8 (1.8)	6 (4.7)		
**Vital signs at presentation**								
Heart rate, bpm	96.0 ± 27.6	97.0 ± 26.0	94.8 ± 27.0	96.5 ± 27.9	94.5 ± 27.8	96.7 ± 28.9	0.27	3488
< 60 beats/min*	232 (6.7)	15 (6.3)	43 (6.9)	131 (6.4)	33 (7.4)	10 (7.9)	0.90	3488
Systolic BP, mmHg	148.3 ± 34.9	142.5 ± 31.5	144.5 ± 34.5	148.9 ± 35.0	153.5 ± 35.5	149.7 ± 36.8	< 0.001	3500
Systolic BP < 90 mm Hg*	87 (2.5)	5 (2.1)	23 (3.7)	50 (2.4)	7 (1.6)	2 (1.6)	0.22	3500
Diastolic BP, mmHg	85.3 ± 23.8	80.0 ± 19.7	83.7 ± 24.6	85.7 ± 23.7	88.0 ± 24.5	86.6 ± 25.5	< 0.001	3494
**Rhythms at presentation**							0.58	3509
Sinus Rhythm	1964 (56.0)	145 (60.9)	355 (56.2)	1140 (55.2)	246 (54.9)	78 (61.4)		
Atrial fibrillation or flutter	1271 (36.2)	76 (31.9)	235 (37.2)	747 (36.2)	172 (38.4)	41 (32.3)		
NYHA class III or IV	3042 (86.9)	216 (90.8)	556 (88.3)	1760 (85.5)	393 (87.9)	117 (92.9)	0.02	3499
**Test results at admission**								
LVEF, %	46.2 ± 16.2	45.6 ± 16.7	45.6 ± 16.3	46.1 ± 16.0	48.3 ± 16.6	45.4 ± 17.3	0.048	3435
LVEF classification							0.27	3498
HFrEF (LVEF < 40%)*	1321 (37.8)	94 (39.5)	239 (37.9)	788 (38.3)	148 (33.2)	52 (41.3)		
HFmrEF (LVEF40%-49%)	659 (18.8)	42 (17.7)	121 (19.2)	400 (19.5)	78 (17.5)	18 (14.3)		
HFpEF (LVEF ≥ 50%)	1518 (43.4)	102 (42.9)	271 (43.0)	869 (42.3)	220 (49.3)	56 (44.4)		
BNP, pg/ml	710 (387–1251)	907 (508–1663)	935 (512–1548)	711 (412–1242)	461 (269–784)	384 (214–777)	< 0.001	3108
NT-proBNP, pg/ml	5416 (2631–11,955)	11,732 (4772–23,213)	8530 (3836–16,947)	5314 (2678–10,746)	4036 (1982–6293)	2388 (1055–5361)	< 0.001	614
Serum creatinine, mg/dl	1.48 ± 1.28	1.24 ± 0.84	1.33 ± 1.04	1.54 ± 1.37	1.52 ± 1.36	1.43 ± 1.06	< 0.001	3503
eGFR, ml/min/1.73m^2^	46.3 ± 23.4	48.6 ± 25.1	48.0 ± 24.9	45.1 ± 22.7	47.1 ± 23.2	48.8 ± 23.9	0.07	3503
< 60 ml/min/1.73m^2^	2588 (73.9)	172 (72.9)	446 (70.7)	1559 (75.6)	320 (71.4)	91 (71.7)	0.07	3503
< 30 ml/min/1.73m^2^*	921 (26.3)	59 (25.0)	165 (26.2)	550 (26.7)	121 (27.0)	26 (20.5)	0.61	3503
Blood urea nitrogen, mg/dl	28.3 ± 16.1	29.6 ± 15.5	29.1 ± 16.0	28.5 ± 16.4	25.6 ± 14.4	26.6 ± 17.7	< 0.001	3498
Albumin, g/dl	3.49 ± 0.49	3.27 ± 0.50	3.39 ± 0.45	3.52 ± 0.48	3.62 ± 0.50	3.56 ± 0.50	< 0.001	3408
< 3.0 g/dl*	438 (12.9)	53 (22.9)	95 (15.5)	239 (12.0)	38 (8.6)	13 (10.7)	< 0.001	3408
Sodium, mEq/l	139.2 ± 4.1	138.7 ± 4.4	138.9 ± 4.7	139.2 ± 4.1	139.8 ± 3.5	139.1 ± 4.2	0.005	3498
< 135 mEq/l*	405 (11.6)	32 (13.6)	102 (16.2)	221 (10.7)	35 (7.8)	15 (11.9)	< 0.001	3498
Hemoglobin, g/dl	11.6 ± 2.4	10.9 ± 2.0	11.1 ± 2.2	11.6 ± 2.3	12.2 ± 2.5	12.7 ± 2.5	< 0.001	3503
Anemia*	2299 (65.6)	180 (75.6)	470 (74.5)	1337 (64.9)	251 (56.0)	61 (48.0)	< 0.001	3503
CRP, mg/dL	1.99 ± 3.58	2.27 ± 3.82	2.06 ± 3.41	2.02 ± 3.75	1.67 ± 2.94	1.72 ± 3.13	0.59	3422
**Medication at discharge**								
ACEI or ARB*	2058 (58.6)	103 (43.3)	335 (53.0)	1227 (59.5)	305 (68.1)	88 (69.3)	< 0.001	3509
β blocker*	2376 (67.7)	125 (52.5)	400 (63.3)	1426 (69.1)	327 (73.0)	98 (77.2)	< 0.001	3509
MRA	1589 (45.3)	114 (47.9)	314 (49.7)	895 (43.4)	196 (43.8)	70 (55.1)	0.007	3509
Loop diuretics	2865 (81.6)	198 (83.2)	520 (82.3)	1678 (81.3)	358 (79.9)	111 (87.4)	0.35	3509
Tolvaptan*	377 (10.7)	17 (7.1)	67 (10.6)	239 (11.6)	34 (7.6)	20 (15.8)	0.01	3509
**Congestion at discharge**								
Edema	419 (12.3)	28 (12.3)	55 (9.1)	248 (12.4)	56 (12.9)	32 (25.4)	< 0.001	3399
Pulmonary congestion	270 (7.8)	23 (9.8)	50 (8.1)	157 (7.7)	25 (5.6)	15 (11.9)	0.12	3457
Jugular venous distention	224 (6.6)	15 (6.6)	37 (6.1)	134 (6.7)	25 (5.8)	13 (10.3)	0.48	3377

Values are number (%), mean ± SD, or median (interquartile range). P values were calculated using the chi square test or Fisher’s exact test for categorical variables, and 1-way ANOVA or Kruskal–Wallis test for continuous variables.

*Risk-adjusting variables selected for the Cox proportional hazard models.

Chronic kidney disease was defined as estimated glomerular filtration rate (eGFR) < 60 mL/min/1.73 m^2^. Renal dysfunction was defined as estimated glomerular filtration rate (eGFR) < 30 mL/min/1.73 m^2^ based on the chronic kidney disease grades.

Anemia was defined using the World Health Organization criteria (hemoglobin < 12.0 g/dl in women and < 13.0 g/dl in men).

*ACEI* angiotensin-converting enzyme inhibitor, *ACS* acute coronary syndrome, *ARB* angiotensin-receptor blocker, *BNP* brain-type natriuretic peptide, *BMI* body mass index, *BP* blood pressure, *eGFR* estimated glomerular filtration rate, *HFmrEF* heart failure with mid-range ejection fraction, *HFpEF* heart failure with preserved ejection fraction, *HFrEF* heart failure with reduced ejection fraction, *LVEF* left ventricular ejection fraction, *NT-pro BNP* N-terminal-pro brain-type natriuretic peptide, *NYHA* New York Heart Association.

### Clinical outcomes

**Figure 2:**
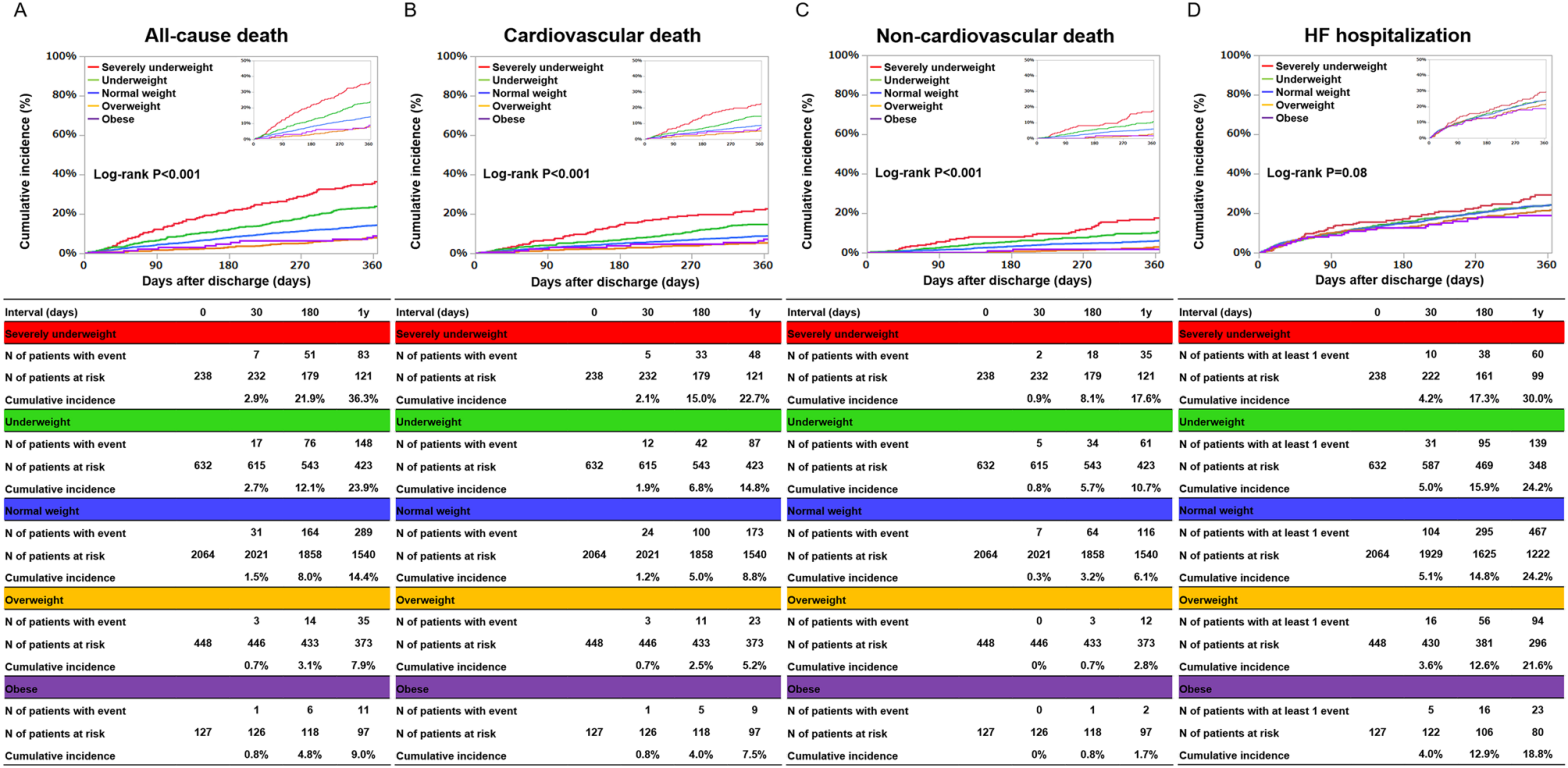
Kaplan–Meier curves for the primary and secondary outcome measures. (**A**) All-cause death (**B**) Cardiovascular death (**C**) Non-cardiovascular death (**D**) HF hospitalization. *HF* heart failure.

**Figure 3:**
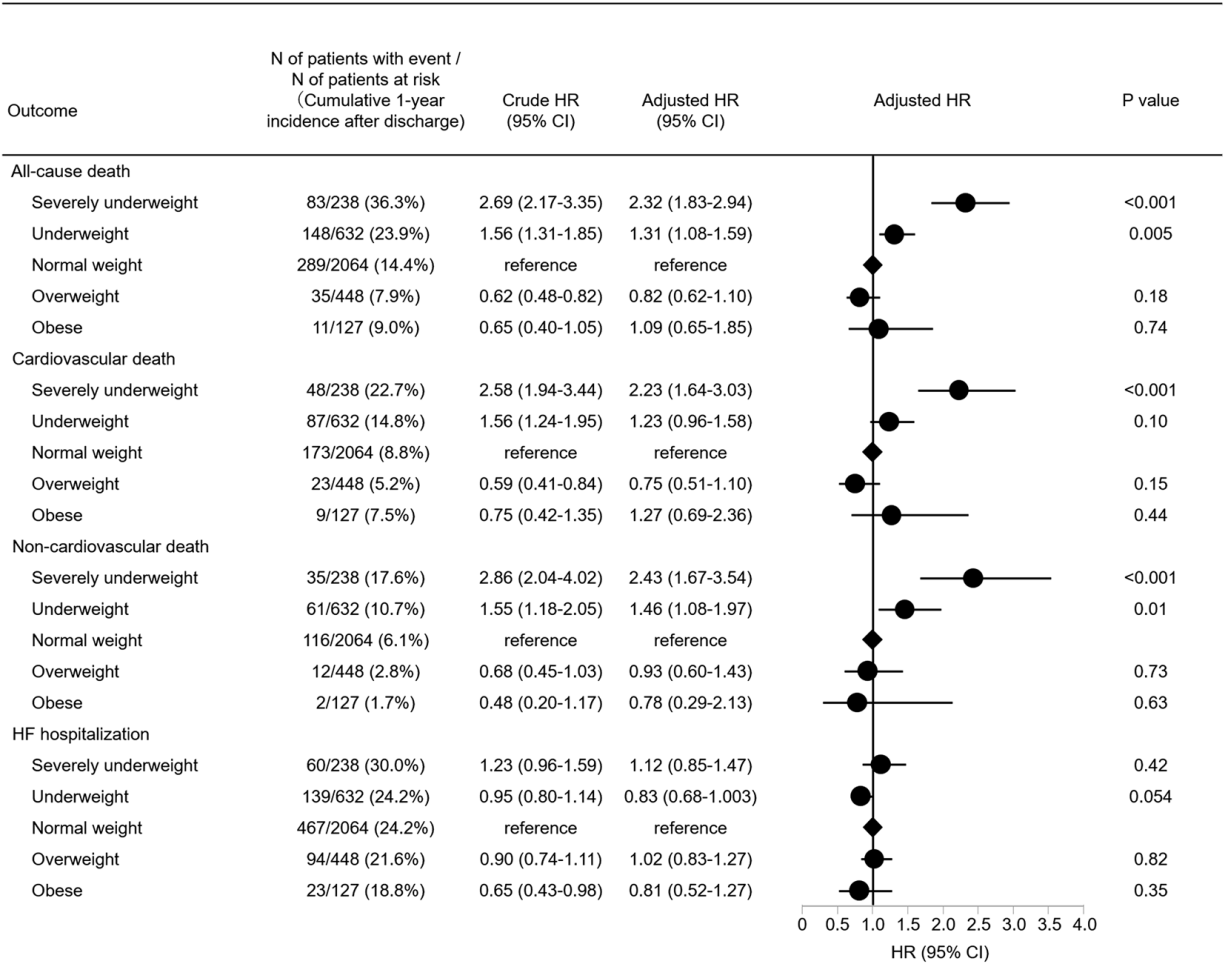
Forrest plots for the adjusted hazard ratios of each BMI category for the clinical outcome measures. *BMI*, body mass index; *CI*, confidence interval; *HF*, heart failure; *HR*, hazard ratio.

The median follow-up duration was 471 (IQR: 378–666) days, with a 96.4% follow up rate at 1 year. The cumulative 1-year incidence of all-cause death was higher in underweight groups, and lower in overweight groups (Severely underweight: 36.3%, Underweight: 23.9%, Normal weight: 14.4%, Overweight: 7.9%, and Obese: 9.0%, *P* < 0.001) (Fig. 2A). After adjusting for confounders, the excess risk for all-cause death remained significant in the severely underweight group (HR, 2.32; 95%CI, 1.83–2.94; *P* < 0.001), and in the underweight group (HR, 1.31; 95%CI, 1.08–1.59; *P* = 0.005) relative to the normal weight group, while the lower risk for all-cause death was no longer significant in the overweight group (HR, 0.82; 95%CI, 0.62–1.10; *P* = 0.18) and the excess risk for all-cause death was no significant in the obese group (HR, 1.09; 95%CI, 0.65–1.85; *P* = 0.74) (Fig. 3). The cumulative 1-year incidence of cardiovascular death was also higher in underweight groups, and lower in overweight groups (Severely underweight: 22.7%, Underweight: 14.8%, Normal weight: 8.8%, Overweight: 5.2%, and Obese: 7.5%, *P* < 0.001) (Fig. 2B). After adjusting for confounders, the excess risk for cardiovascular death remained significant in the severely underweight group (HR, 2.23; 95%CI, 1.64–3.03; *P* < 0.001), relative to the normal weight group, while the excess risk for cardiovascular death was no longer significant in the underweight group (HR, 1.23; 95%CI, 0.96–1.58; *P* = 0.10) and the obese group (HR, 1.27; 95%CI, 0.69–2.36; *P* = 0.44) and the lower risk for cardiovascular death was no longer significant in the overweight group (HR, 0.75; 95%CI, 0.51–1.10; *P* = 0.15) (Fig. 3). The cumulative 1-year incidence of non-cardiovascular death was also higher in underweight groups, and lower in overweight groups (Severely underweight: 17.6%, Underweight: 10.7%, Normal weight: 6.1%, Overweight: 2.8%, and Obese: 1.7%, *P* < 0.001) (Fig. 2C). After adjusting for confounders, the excess risk for non-cardiovascular death remained significant in the severely underweight group (HR, 2.43; 95%CI, 1.67–3.54; *P* < 0.001), and in the underweight group (HR, 1.46; 95%CI, 1.08–1.97; *P* = 0.01) relative to the normal weight group, while the lower risk for non-cardiovascular death was no longer significant in the overweight group (HR, 0.93; 95%CI, 0.60–1.43; *P* = 0.73), and in the obese group (HR, 0.78; 95%CI, 0.29–2.13; *P* = 0.63) (Fig. 3). The cumulative 1-year incidence of HF hospitalization decreased with increasing BMI (Severely underweight: 30.0%, Underweight: 24.2%, Normal weight: 24.2%, Overweight: 21.6%, and Obese: 18.8%, *P* < 0.001) (Fig. 2D). After adjusting for confounders, the risk for HF hospitalization was not significantly different across the 5 groups stratified by BMI (Fig. 3).

### Subgroup analysis

**Figure 4:**
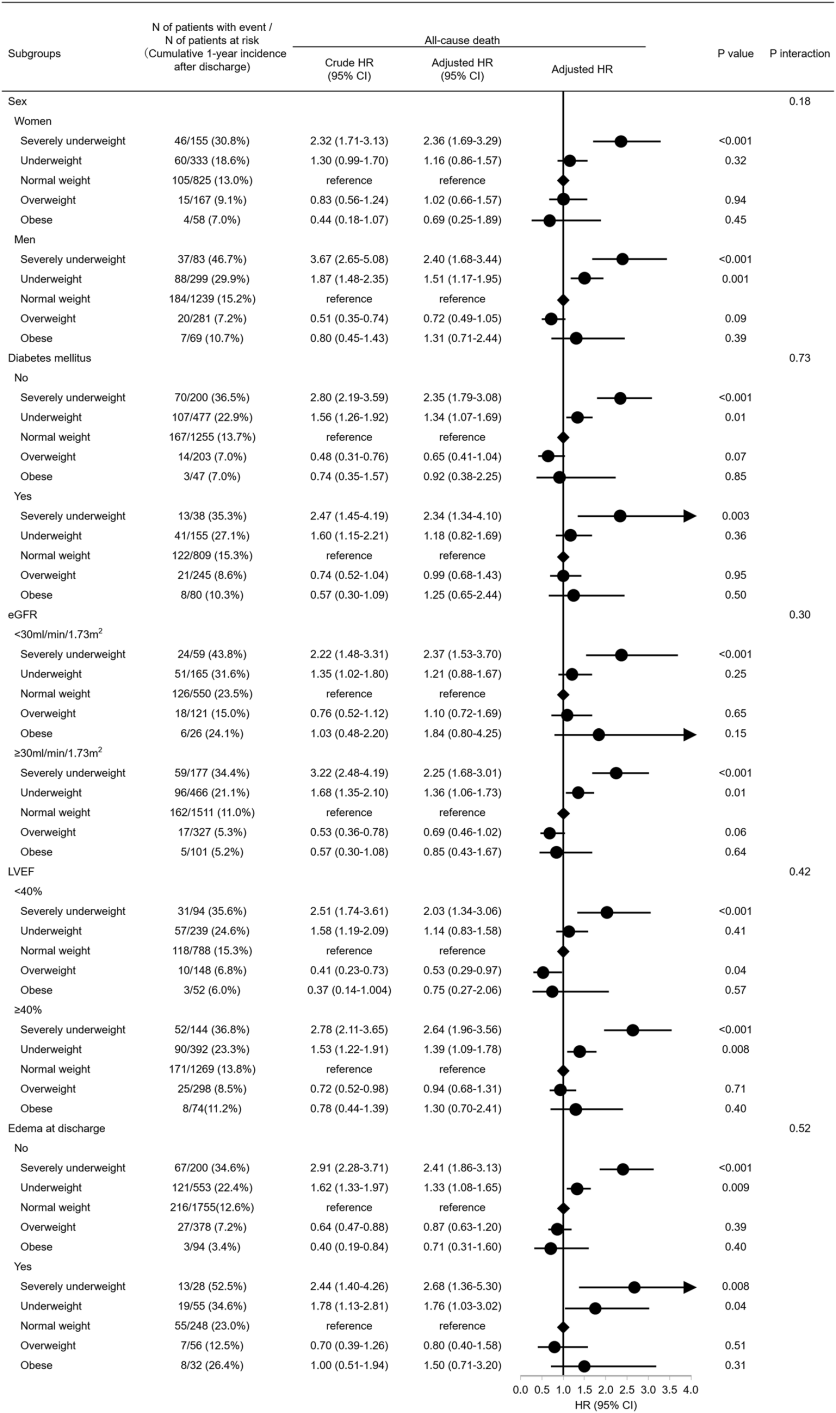
Subgroup analyses for the primary outcome measure (all-cause death). *BMI* body mass index, *CI* confidence interval, *eGFR* estimated glomerular filtration rate, *HR* hazard ratio, *LVEF* left ventricular ejection fraction.

The BMI at discharge was significantly lower in the subgroups of women, and patients without edema at discharge than those without (Supplementary Table 2). There was no significant interaction between the subgroup factors and the effect of the each BMI group relative to normal weight group on all-cause death (Fig. 4).

### Sensitivity analysis using modified classification for Asian populations

When we used the modified classification in which the 16, 18.5, 23, and 27.5 kg/m^2^ cutoffs were used (Supplementary Table 3), the results (Supplementary Figs. 2 and 3) were mostly consistent with the main analysis. BMIs of < 16 kg/m^2^ were associated with increased risk, whereas BMIs of ≥ 23 kg/m^2^ but < 27.5 kg/m^2^ were associated with decreased risk of all-cause death and cardiovascular death in patients as compared with BMIs of ≥ 18.5 kg/m^2^ but < 23 kg/m^2^ (Supplementary Figs. 2 and 3).

### Additional analysis using BMI quartiles at discharge

When we divided the participants into BMI quartiles (Supplementary Table 4), the results (Supplementary Figs. 4 and 5) were mostly consistent with the main analysis. The lowest quartile was associated with increased risk of all-cause death, cardiovascular death, and non-cardiovascular death in patients as compared with BMIs of ≥ 20.9 kg/m^2^ but < 23.6 kg/m^2^ (Supplementary Figs. 4 and 5). Highest quartile (BMI ≥ 23.6 kg/m^2^) was not associated with increased or decreased risks of all-cause death, cardiovascular death, and non-cardiovascular death in patients as compared with BMIs of ≥ 20.9 kg/m^2^ but < 23.6 kg/m^2^ (Supplementary Figs. 4 and 5).

### Prognostic implications of BMI at admission

When we stratified the patients into 5 groups according to BMI at admission based on the WHO classification (Supplementary Table 5, Supplementary Fig. 6), the results were mostly consistent with the main analysis. The excess risk for all-cause death remained significant in the severely underweight group and in the underweight group, whereas overweight was associated with decreased risk of all-cause death and cardiovascular death in patients compared to normal weight (Supplementary Figs. 7 and 8).

## Discussion

The main findings of this study were as follows; (1) Lower BMI, especially severely underweight status, was associated with increased mortality in patients after discharge with HF; (2) Overweight and obesity based on WHO classifications were not associated with increased or decreased risk of death in patients compared to normal weight status; (3) The risk for HF hospitalization was not affected by BMI status.

The association of BMI and prognosis in patients with HF has long been investigated. However, there is only one report on the prognostic significance of a severely underweight status in patients with HF. Matsushita et al. reported a severely low BMI was associated with mortality in the patients with ADHF, but the number of patients with a severely underweight BMI were limited^22^ and the risk for death compared to that of normal weight status was unclear^22^. Using the large database in Japan, we showed that the severely underweight status was associated with all-cause, cardiovascular, and non-cardiovascular death. Our results are consistent with previous studies, which have shown a lower BMI is associated with a higher risk of death^4,5,7,8,23^. The classification of BMI did not influence the risk of hospitalization for HF in multivariable analyses, which is consistent with the results of the DIG and CHARM sub-studies^5,23^. The 1-year mortality after hospital discharge for ADHF is relatively low in the present study and ranged from 16.5% (cumulative 1-year mortality) to 22.2% in other Japanese studies^24^ as compared with that in the United States^25^, despite that older patients were enrolled in the Japanese registries. This might be due to the differences in ethnicity, HF etiology, and enrollment timeframe. Despite these differences, the prognostic influence of low BMI was observed across studies worldwide.

The mechanistic link between underweight status and poor outcome in patients with HF has been proposed. A lower BMI reflects a decrease in skeletal muscle, implying the associated malnutrition and inflammation^17,26,27^. In fact, both the LVEF and NYHA status at presentation were not different among the BMI statuses. The albumin and hemoglobin levels was incrementally lower in the underweight groups. A reduction in food intake, gastrointestinal abnormalities, immunological and neurohormonal activation as well as an imbalance between anabolic and catabolic processes may be important mechanisms to understand these conditions^26–29^. After adjusting for confounders such as age, sex, and the presence of anemia, the association between being underweight and a poor prognosis remained significant.

In our study, the mortality was lowest in patients with an overweight status, followed by those with an obese status, although there was no significant difference from patients with a normal weight status based on the WHO classifications^30,31^. The patients in the overweight and obese groups were younger, had more metabolic diseases and decreased levels of BNP, and were more likely to be administered with an ACE-I/ARB or β-blocker. Low mortality rates in patients with higher BMI might be related to a greater metabolic reserve against stress^32^, a reduced cardiac sympathetic activity^33^, an attenuated neurohormonal response^34^, and a lower inflammatory cytokine levels, and lesser catabolic-anabolic imbalance^35^.

In the theory of obesity paradox, having a larger BMI is associated with better outcomes; however, many of previous studies stratified patients into two groups for comparisons^36–38^. In other studies, risk for all-cause death was lowest in obese patients (BMI ≥ 30 kg/m^2^)^2,7^. In contrast, a sub-study of CHARM trial reported that patients with BMI ≥ 35 kg/m^2^ tended to show a worse prognosis^5^. Nagarajan et al. from Cleveland Clinic HF program demonstrated a poor prognosis in very obese patients (BMI ≥ 40 kg/m^2^) with advanced HF^39^. In the present study, the effect of higher BMI on mortality was inconclusive mainly due to small number of patients with higher BMI. The prevalence of overweight and obese HF patients was much lower than reports in previous studies based on randomized trials in Western countries^5^ and previous studies from Japan conducted in 2004^40^ and 2007^36^. The differences in patient backgrounds may be derived from the style of the study and the countries and periods of enrollment, focusing on the increase in aging patients with ADHF. The risk of all-cause death in obese patients was also inconclusive, but that in overweight patients became significant when we adopted the cutoffs for the Asian population. In Japan, the cutoff BMI is authorized by the guidelines of the Japan Society for the Study of Obesity and is basically identical to the WHO classification^30,31^. Defining the ideal BMI values in Japanese patient with ADHF is beyond the scope of the present study, and further studies are required to validate the cutoff BMI in Japan. Ideal body weight in patients with HF should be set individually and we should take ethnicity as well as comorbidities in consideration. Admission BMI was also associated with prognosis, even recognizing the setback of congestion. This result was consistent with the subgroup analysis stratified with or without edema at discharge. Considering the prognostic impact of BMI at admission and discharge, the evaluation of BMI is always critically important for the assessment of patients with ADHF. However, the BMI at admission can be more easily changed through the treatment for ADHF. In the present study, the mean difference between the BMIs at admission and discharge was 1.5 kg/m^2^ (Table 1). Thus, the BMI at discharge would be a more reliable marker for patients with ADHF. Our study will be useful to understand the pathophysiology of ADHF and patients’ conditions, and to evaluate the prognosis of patients with ADHF. When the BMI of a given patient is severely low, special attention should be paid to the worsening of HF and non-cardiovascular diseases. Future research would be warranted to identify and promote achieving the optimal BMI in individual patients.

### Limitations

This study had several limitations. First, we could not determine the body weight at discharge was an optimal body weight without congestion in a given patient. Although the body weight at discharge was decreased compared to that at admission, a substantial proportion of patients had residual edema at discharge. Second, serum levels of cytokines, catecholamines, and renin and aldosterone were not collected. Thus, we can only speculate on the mechanistic link between the low BMI and poor outcome based on the available data in the present study. Third, residual unmeasured confounding factors could affect the results even after extensive adjustment. Due to lots of potential confounders, the conclusion should be treated with caution. Fourth, several subgroup analyses have a risk for multiple comparisons as well as a small sample size with low statistical power. Fifth, a selection bias might have been present. The patients with unavailable BMI data included older patients with anemia and hypoalbuminemia, and low ambulatory status. The lack of BMI data may be due to the patients’ non-ambulatory status. The characteristics of the patients with unavailable BMI data were similar to those of underweight or severely underweight patients. The non-ambulatory patients showed worse outcomes^16^; thus, excluding these patients may not change the result of this study. Sixth, owing to the short-term follow-up, the causal link between BMI and outcome is unclear. Further research studies are needed to clarify the causal link.

## Conclusion

Very low BMI was associated with a higher risk for one-year mortality after discharge in patients with ADHF.

## Supplementary information

Supplementary Information.
