# Supplementary material for: Association between body mass index and prognosis of patients hospitalized with heart failure
Source: Sci Rep. 2020 Oct 7;10:16663. doi: 10.1038/s41598-020-73640-w (PMC7542148; doi:10.1038/s41598-020-73640-w)

**Supplementary Information for**

**Association between Body Mass Index and Prognosis of Patients Hospitalized With Heart Failure**

**Authors:** Yuta Seko, Takao Kato, Takeshi Morimoto, Hidenori Yaku, Yasutaka Inuzuka, Yodo Tamaki, Neiko Ozasa, Masayuki Shiba, Erika Yamamoto, Yusuke Yoshikawa, Yugo Yamashita, Takeshi Kitai, Ryoji Taniguchi, Moritake Iguchi, Kazuya Nagao, Takafumi Kawai, Akihiro Komasa, Ryusuke Nishikawa, Yuichi Kawase, Takashi Morinaga, Mamoru Toyofuku, Yutaka Furukawa, Kenji Ando, Kazushige Kadota, Yukihito Sato, Koichiro Kuwahara, Takeshi Kimura.

**Supplementary Information content**

**Supplementary Methods Page 3-5**

**References for supplementary materials Page 5**

**Supplementary Tables Page 6-23**

**Supplementary Figure Legends Page 24-25**

**Supplementary Figures Page 26-33**

**Supplementary Methods**

**Sensitivity analysis using modified classification for Asian populations**

We divided the participants into 5 groups using the 16, 18.5, 23 and 27.5 Kg/m^2^ cut-offs [1,2]; (1) BMI <16 kg/m^2^, (2) BMI ≥ 16 kg/m^2^ and < 18.5 kg/m^2^, (3) BMI ≥18.5 kg/m^2^ and < 23 kg/m^2^, (4) BMI ≥ 23 kg/m^2^ and < 27.5 kg/m^2^, and (5) BMI ≥ 27.5 kg/m^2^ (Supplementary Table 3). We estimated the cumulative incidences of the clinical events during 1-year after discharge using the Kaplan-Meier method with the intergroup differences assessed by the log-rank test (Supplementary Figure 2). To estimate the risk of each BMI group with BMIs of ≥23 kg/m^2^ but <27.5 kg/m^2^ group as the reference, a multivariable Cox proportional hazards model was developed for the primary and secondary outcome measures adjusting for the confounders. (Supplementary Figure 3). We included the same risk-adjusting variables for main analysis into the model.

**Additional analysis using BMI quartiles at discharge**

We divided the participants into BMI quartiles; (1) BMI <18.5 kg/m^2^, (2) BMI ≥ 18.5 kg/m^2^ and < 20.9 kg/m^2^, (3) BMI ≥20.9 kg/m^2^ and < 23.6 kg/m^2^, (4) BMI ≥ 23.6 kg/m^2^ (Supplementary Table 4). We estimated the cumulative incidences of the clinical events during 1-year after discharge using the Kaplan-Meier method with the intergroup differences assessed by the log-rank test (Supplementary Figure 4). To estimate the risk of each BMI group with BMIs of ≥20.9 kg/m^2^ but <23.6 kg/m^2^ group as the reference, a multivariable Cox proportional hazards model was developed for the primary and secondary outcome measures adjusting for the confounders. (Supplementary Figure 5). We included the same risk-adjusting variables for main analysis into the model.

**Additional analysis using BMI classification at admission**

When we stratified the patients into 5 groups according to BMI at admission based on the WHO classification; (1) Severely underweight: BMI <16 kg/m^2^, (2) Underweight: BMI ≥ 16 kg/m^2^ and < 18.5 kg/m^2^, (3) Normal weight: BMI ≥18.5 kg/m^2^ and < 25 kg/m^2^, (4) Overweight: BMI ≥ 25 kg/m^2^ and < 30 kg/m^2^, and (5) Obese: BMI ≥ 30 kg/m^2^ (Supplementary Table 5, Supplementary Figure 6). We estimated the cumulative incidences of the clinical events during 1-year after discharge using the Kaplan-Meier method with the intergroup differences assessed by the log-rank test (Supplementary Figure 7). To estimate the risk of each BMI group with normal weight group as the reference, a multivariable Cox proportional hazards model was developed for the primary and secondary outcome measures adjusting for the confounders (Supplementary Figure 8). We changed risk-adjusting variables at discharge (medication at discharge) to variables at admission: age as a continuous variable, sex, LVEF <40% by echocardiography, variables related to medical history (etiology of HF hospitalization associated with acute coronary syndrome, previous HF hospitalization, atrial fibrillation or flutter, hypertension, diabetes mellitus, previous myocardial infarction, previous stroke, current smoking, chronic lung disease and malignant neoplasm), variables related to comorbidities (living alone, ambulatory, systolic blood pressure <90 mmHg, heart rate <60 bpm, eGFR <30 ml/min/1.73m^2^, albumin <3.0 g/dL, sodium <135 mEq/L, and anemia), and medications at admission (angiotensin converting enzyme inhibitors [ACEIs] or angiotensin II receptor blockers [ARBs], β-blockers, and tolvaptan).

**References for supplementary materials**

[1] Appropriate body-mass index for Asian populations and its implications for policy and intervention strategies. Lancet (London, England). 2004;363:157-63.

[2] Physical status: the use and interpretation of anthropometry. Report of a WHO Expert Committee. World Health Organization technical report series. 1995;854:1-452.

**Supplementary Table 1: Baseline characteristics of the patients available with BMI data and those unavailable with BMI data at discharge**

| **Variable** | **Available BMI data**  **(N=3509)** | **Unavailable BMI data**  **(N=228)** | **P value** |
| --- | --- | --- | --- |
| **Clinical Characteristic** |  |  |  |
| Age, years | 77.2±12.0 | 84.1±9.6 | <0.001 |
| Women | 1538 (43.8) | 137 (60.1) | <0.001 |
| Associated with ACS at admission | 194 (5.5) | 13 (5.7) | 0.91 |
| Heart failure hospitalization | 1272 (36.8) | 51 (24.1) | <0.001 |
| Hypertension | 2551 (72.7) | 156 (68.4) | 0.16 |
| Diabetes mellitus | 1327 (37.8) | 75 (32.9) | 0.14 |
| Atrial fibrillation or flutter | 1477 (42.1) | 81 (35.5) | 0.051 |
| Previous myocardial infarction | 806 (23.0) | 36 (15.8) | 0.01 |
| Previous stroke | 548 (15.6) | 47 (20.6) | 0.046 |
| Current smoking | 442 (12.8) | 11 (5.0) | <0.001 |
| Chronic lung disease | 463 (13.2) | 26 (11.4) | 0.44 |
| Malignancy | 507 (14.5) | 32 (14.0) | 0.86 |
| Living alone | 755 (21.5) | 45 (19.8) | 0.53 |
| Ambulatory | 2837 (81.6) | 118 (52.9) | <0.001 |
| Heart rate, bpm | 96.0±27.6 | 94.6±28.6 | 0.58 |
| <60 beats/min | 232 (6.7) | 21 (9.4) | 0.12 |
| Systolic BP, mmHg | 148.3±34.9 | 150.0±35.6 | 0.40 |
| Systolic BP <90 mm Hg | 87 (2.5) | 8 (3.5) | 0.34 |
| Diastolic BP, mmHg | 85.3±23.8 | 82.4±24.4 | 0.08 |
| LVEF, % | 46.2±16.2 | 48.3±15.6 | 0.08 |
| eGFR<30 mL/min/1.73m^2^ | 921 (26.3) | 67 (29.4) | 0.30 |
| Albumin <3.0 g/dl | 438 (12.9) | 45 (20.6) | 0.001 |
| Sodium <135 mEq/l | 405 (11.6) | 32 (14.2) | 0.24 |
| Anemia, (%) | 2299 (65.6) | 169 (74.5) | 0.007 |
| ACEI or ARB at discharge | 2058 (58.7) | 89 (39.0) | <0.001 |
| β blocker at discharge | 2376 (67.7) | 101 (44.3) | <0.001 |
| Tolvaptan at discharge | 377 (10.7) | 15 (6.6) | 0.047 |

Values are number (%), mean ± SD, or median (interquartile range). P values were calculated using the chi square test or Fisher’s exact test for categorical variables, and the Student’s t test or Wilcoxon rank sum test for continuous variables.

Renal dysfunction was defined as estimated glomerular filtration rate (eGFR) <30 mL/min/1.73 m^2^ based on the chronic kidney disease grades. Anemia was defined using the World Health Organization criteria (hemoglobin <12.0 g/dL in women and <13.0 g/dL in men).

Abbreviations: ACEI, angiotensin-converting enzyme inhibitor; ACS, acute coronary syndrome; ARB, angiotensin-receptor blocker; BP, blood pressure; eGFR, estimated glomerular filtration rate; LVEF, left ventricular ejection fraction

**Supplementary Table 2. BMI at discharge in each subgroup**

| **Subgroup** | **N of patients** | **BMI at discharge** | **P value** |
| --- | --- | --- | --- |
| **Sex** |  |  |  |
| Women | 1538 | 20.8±4.3 | <0.001 |
| Men | 1971 | 21.8±4.0 |  |
| **Diabetes mellitus** |  |  |  |
| No | 2182 | 20.6±3.8 | <0.001 |
| Yes | 1327 | 22.6±4.5 |  |
| **eGFR <30 ml/min/1.73m^2^** |  |  |  |
| No | 2582 | 21.4±4.3 | 0.31 |
| Yes | 921 | 21.2±3.8 |  |
| **LVEF, %** |  |  |  |
| <40 | 1321 | 21.3±4.3 | 0.45 |
| ≥40 | 2177 | 21.4±4.2 |  |
| **Edema at discharge** |  |  |  |
| No | 2980 | 21.3±4.1 | 0.003 |
| Yes | 419 | 22.1±4.6 |  |

BMI value are presented as the mean ± SD and were compared using the Wilcoxon’s rank sum test.

BMI, body mass index; eGFR, estimated glomerular filtration rate; LVEF, left ventricular ejection fraction.

**Supplementary Table 3. Baseline characteristics of the study subjects and transthoracic echocardiography results of the patients in modified classification for Asian populations**

| **Variables** | **Total**  **(N=3509)** | **BMI<16**  **(N=238)** | **16≤BMI<18.5 (N=632)** | **18.5≤BMI<23 (N=1590)** | **23≤BMI<27.5 (N=802)** | **27.5≤BMI (N=247)** | **P value** | **Total N** |
| --- | --- | --- | --- | --- | --- | --- | --- | --- |
| **Clinical Characteristic** |  |  |  |  |  |  |  |  |
| Age*, years | 77.2±12.0 | 83.2±9.5 | 79.9±10.7 | 78.2±10.9 | 74.5±12.1 | 67.2±15.9 | <0.001 | 3509 |
| Age ≥80 years | 1770 (50.4) | 179 (75.2) | 374 (59.2) | 834 (52.5) | 319 (39.8) | 64 (25.9) | <0.001 | 3509 |
| Women* | 1538 (43.8) | 155 (65.1) | 333 (52.7) | 650 (40.9) | 295 (36.8) | 105 (42.5) | <0.001 | 3509 |
| Body weight, kg at admission | 56.7±14.6 | 39.6±6.9 | 45.9±7.8 | 54.7±9.0 | 65.7±10.8 | 83.9±18.5 | <0.001 | 3481 |
| BMI at admission | 22.9±4.5 | 16.6±1.9 | 19.1±1.8 | 22.2±1.9 | 26.2±2.1 | 33.0±4.7 | <0.001 | 3481 |
| Body weight, kg at discharge | 52.8±13.5 | 35.3±4.8 | 41.8±5.7 | 51.0±7.5 | 62.1±8.8 | 79.4±16.2 | <0.001 | 3509 |
| BMI at discharge | 21.4±4.2 | 14.9±0.9 | 17.4±0.7 | 20.7±1.3 | 24.8±1.2 | 31.2±3.9 | <0.001 | 3509 |
| **Etiology** |  |  |  |  |  |  | <0.001 | 3509 |
| Ischemic | 1147 (32.7) | 54 (22.7) | 178 (28.2) | 523 (32.9) | 308 (38.4) | 84 (34.0) |  |  |
| Associated with ACS* | 194 (5.5) | 6 (2.5) | 22 (3.5) | 98 (6.2) | 52 (6.5) | 16 (6.5) |  |  |
| Not associated with ACS | 953 (27.2) | 48 (20.2) | 156 (24.7) | 425 (26.7) | 256 (31.9) | 68 (27.5) |  |  |
| Hypertensive | 870 (24.8) | 58 (24.4) | 123 (19.5) | 402 (25.3) | 212 (26.4) | 75 (30.4) |  |  |
| Valvular heart disease | 683 (19.5) | 71 (29.8) | 173 (27.4) | 302 (19.0) | 108 (13.5) | 29 (11.7) |  |  |
| Cardiomyopathy | 534 (15.2) | 33 (13.9) | 103 (16.3) | 235 (14.8) | 122 (15.2) | 41 (16.6) |  |  |
| Dilated cardiomyopathy | 386 (11.0) | 23 (9.7) | 69 (10.9) | 166 (10.4) | 95 (11.9) | 33 (13.4) |  |  |
| Arrhythmia-related | 164 (4.7) | 12 (5.0) | 32 (5.1) | 77 (4.8) | 37 (4.6) | 6 (2.4) |  |  |
| **Medical history** |  |  |  |  |  |  |  |  |
| Heart failure hospitalization* | 1272 (36.8) | 101 (42.6) | 247 (39.5) | 568 (36.4) | 262 (33.2) | 94 (38.7) | 0.04 | 3456 |
| Hypertension* | 2551 (72.7) | 151 (63.5) | 411 (65.0) | 1167 (73.4) | 631 (78.7) | 191 (77.3) | <0.001 | 3509 |
| Diabetes* | 1327 (37.8) | 38 (16.0) | 155 (24.5) | 599 (37.7) | 387 (48.3) | 148(59.9) | <0.001 | 3509 |
| Dyslipidemia | 1393 (39.7) | 65 (27.3) | 197 (31.2) | 613 (38.6) | 381 (47.5) | 137 (55.5) | <0.001 | 3509 |
| Atrial fibrillation or flutter* | 1477 (42.1) | 104 (43.7) | 272 (43.0) | 661 (41.6) | 344 (42.9) | 96 (38.9) | 0.75 | 3509 |
| VT/VF | 147 (4.2) | 6 (2.5) | 29 (4.6) | 64 (4.0) | 30 (3.7) | 18 (7.3) | 0.08 | 3509 |
| Previous myocardial infarction* | 806 (23.0) | 38 (16.0) | 134 (21.2) | 371 (23.3) | 204 (25.4) | 59 (23.9) | 0.03 | 3509 |
| Prior PCI or CABG | 924 (26.3) | 42 (17.7) | 141 (22.3) | 416 (26.2) | 246 (30.7) | 79 (32.0) | <0.001 | 3509 |
| Previous stroke* | 548 (15.6) | 32 (13.5) | 106 (16.8) | 255 (16.0) | 128 (16.0) | 27 (10.9) | 0.21 | 3509 |
| Current smoking* | 442 (12.8) | 25 (10.6) | 63 (10.2) | 185 (11.9) | 124 (15.7) | 45 (18.4) | <0.001 | 3450 |
| Chronic lung disease* | 463 (13.2) | 34 (14.3) | 93 (14.7) | 192 (12.1) | 111 (13.8) | 33 (13.4) | 0.47 | 3509 |
| COPD | 289 (8.2) | 27 (11.3) | 70 (11.1) | 124 (7.8) | 56 (7.0) | 12 (4.9) | 0.004 | 3509 |
| Liver cirrhosis | 46 (1.3) | 0 (0) | 3 (0.5) | 27 (1.7) | 12 (1.5) | 4 (1.6) | 0.07 | 3509 |
| Malignancy * | 507 (14.4) | 45 (18.9) | 109 (17.3) | 230 (14.5) | 98 (12.2) | 25 (10.1) | 0.005 | 3509 |
| Dementia | 569 (16.2) | 69 (29.0) | 131 (20.7) | 273 (17.2) | 78 (9.7) | 18 (7.3) | <0.001 | 3509 |
| **Social background on admission** |  |  |  |  |  |  |  |  |
| Poor medical adherence | 586 (16.7) | 35 (14.7) | 113 (17.9) | 250 (15.7) | 149 (18.6) | 39 (15.8) | 0.33 | 3509 |
| Living alone* | 755 (21.5) | 50 (21.0) | 140 (22.2) | 331 (20.8) | 170 (21.2) | 64 (25.9) | 0.47 | 3509 |
| Employed | 484 (13.8) | 11 (4.6) | 60 (9.5) | 179 (11.3) | 161 (20.1) | 73(29.6) | <0.001 | 3509 |
| Public financial assistance | 207 (5.9) | 13 (5.5) | 39 (6.2) | 88 (5.5) | 49 (6.1) | 18 (7.3) | 0.83 | 3509 |
| **Daily life activities** |  |  |  |  |  |  | <0.001 | 3475 |
| Ambulatory* | 2837 (81.6) | 161 (68.2) | 475 (76.2) | 1306 (83.0) | 682 (85.7) | 213 (86.2) |  |  |
| Use of wheelchair (outdoor only) | 251 (7.2) | 16 (6.8) | 53 (8.5) | 122 (7.8) | 46 (5.8) | 14 (5.7) |  |  |
| Use of wheelchair (outdoor and indoor) | 297 (8.5) | 45 (19.1) | 72 (11.6) | 116 (7.4) | 52 (6.5) | 12 (4.9) |  |  |
| Bedridden | 90 (2.6) | 14 (5.9) | 23 (3.7) | 29 (1.8) | 16 (2.0) | 8 (3.2) |  |  |
| **Vital signs at presentation** |  |  |  |  |  |  |  |  |
| Heart rate, bpm | 96.0±27.6 | 97.0±26.0 | 94.8±27.0 | 95.6±27.3 | 97.3±29.0 | 96.2±28.0 | 0.40 | 3488 |
| <60 beats/min* | 232 (6.7) | 15 (6.3) | 43 (6.9) | 104 (6.6) | 50 (6.3) | 20 (8.2) | 0.87 | 3488 |
| Systolic BP, mmHg | 148.3±34.9 | 142.5±31.5 | 144.5±34.5 | 148.5±35.2 | 151.7±35.0 | 150.8±35.9 | <0.001 | 3500 |
| Systolic BP <90 mm Hg* | 87 (2.5) | 5 (2.1) | 23 (3.7) | 43 (2.7) | 12 (1.5) | 4 (1.6) | 0.09 | 3500 |
| Diastolic BP, mmHg | 85.3±23.8 | 80.0±19.7 | 83.7±24.6 | 84.9±23.6 | 88.0±24.0 | 87.8±25.2 | <0.001 | 3494 |
| **Rhythms at presentation** |  |  |  |  |  |  | 0.11 | 3509 |
| Sinus Rhythm | 1964 (56.0) | 145 (60.9) | 355 (56.2) | 867 (54.5) | 451 (56.2) | 146 (59.1) |  |  |
| Atrial fibrillation or flutter | 1271 (36.2) | 76 (31.9) | 235 (37.2) | 573 (36.0) | 301 (37.5) | 86 (34.8) |  |  |
| NYHA class III or IV | 3042 (86.9) | 216 (90.8) | 556 (88.3) | 1350 (85.1) | 697 (87.3) | 223 (90.7) | 0.02 | 3499 |
| **Test results at admission** |  |  |  |  |  |  |  |  |
| LVEF, % | 46.2±16.2 | 45.6±16.7 | 45.6±16.3 | 46.0±16.1 | 47.3±16.0 | 46.3±17.1 | 0.33 | 3435 |
| LVEF classification |  |  |  |  |  |  | 0.88 | 3498 |
| HFrEF (LVEF<40%)* | 1321 (37.8) | 94 (39.5) | 239 (37.9) | 603 (38.1) | 291 (36.4) | 94 (38.2) |  |  |
| HFmrEF (LVEF40%-49%) | 659 (18.8) | 42 (17.7) | 121 (19.2) | 307 (19.4) | 151 (18.9) | 38 (15.5) |  |  |
| HFpEF (LVEF≥50%) | 1518 (43.4) | 102 (42.9) | 271 (43.0) | 673 (42.5) | 358 (44.8) | 114(46.3) |  |  |
| BNP, pg/ml | 710 (387-1251) | 907 (508-1663) | 935 (512-1548) | 752 (446-1331) | 530 (293-916) | 411 (222-717) | <0.001 | 3108 |
| NT-proBNP, pg/ml | 5416 (2631-11955) | 11732 (4772-23213) | 8530 (3836-16947) | 5738 (2940-11110) | 4036 (2260-7887) | 2938 (1316-5423) | <0.001 | 614 |
| Serum creatinine, mg/dl | 1.48±1.28 | 1.24±0.84 | 1.33±1.04 | 1.55±1.37 | 1.51±1.34 | 1.50±1.29 | <0.001 | 3503 |
| eGFR, ml/min/1.73m^2^ | 46.3±23.4 | 48.6±25.1 | 48.0±24.9 | 44.8±22.6 | 46.6±22.9 | 47.9±23.7 | 0.06 | 3503 |
| <60 ml/min/1.73m^2^ | 2588 (73.9) | 172 (72.9) | 446 (70.7) | 1208 (76.1) | 588 (73.4) | 174 (70.5) | 0.06 | 3503 |
| <30 ml/min/1.73m^2^* | 921 (26.3) | 59 (25.0) | 165 (26.2) | 436 (27.5) | 202 (25.2) | 59 (23.9) | 0.64 | 3503 |
| Blood urea nitrogen, mg/dl | 28.3±16.1 | 29.6±15.5 | 29.1±16.0 | 29.1±16.7 | 26.3±14.9 | 25.9±16.0 | <0.001 | 3498 |
| Albumin, g/dl | 3.49±0.49 | 3.27±0.50 | 3.39±0.45 | 3.50±0.48 | 3.60±0.49 | 3.60±0.49 | <0.001 | 3408 |
| <3.0 g/dl* | 438 (12.9) | 53 (22.9) | 95 (15.5) | 198 (12.8) | 71 (9.1) | 21 (8.7) | <0.001 | 3408 |
| Sodium, mEq/l | 139.2±4.1 | 138.7±4.4 | 138.9±4.7 | 139.2±4.1 | 139.4±3.9 | 139.6±3.6 | 0.048 | 3498 |
| <135 mEq/l* | 405 (11.6) | 32 (13.6) | 102 (16.2) | 179 (11.3) | 70 (8.8) | 22 (8.9) | <0.001 | 3498 |
| Hemoglobin, g/dl | 11.6±2.4 | 10.9±2.0 | 11.1±2.2 | 11.5±2.3 | 12.1±2.5 | 12.5±2.5 | <0.001 | 3503 |
| Anemia* | 2299 (65.6) | 180 (75.6) | 470 (74.5) | 1067 (67.3) | 455 (56.8) | 127 (51.4) | <0.001 | 3503 |
| CRP, mg/dL | 1.99±3.58 | 2.27±3.82 | 2.06±3.41 | 2.05±3.78 | 1.82±3.36 | 1.69±3.06 | 0.56 | 3422 |
| **Medication at discharge** |  |  |  |  |  |  |  |  |
| ACEI or ARB* | 2058 (58.6) | 103 (43.3) | 335 (53.0) | 928 (58.4) | 521 (65.0) | 171 (69.2) | <0.001 | 3509 |
| β blocker* | 2376 (67.7) | 125 (52.5) | 400 (63.3) | 1081 (68.0) | 584 (72.8) | 186 (75.3) | <0.001 | 3509 |
| MRA | 1589 (45.3) | 114 (47.9) | 314 (49.7) | 684 (43.0) | 360 (44.9) | 117 (47.4) | 0.053 | 3509 |
| Loop diuretics | 2865 (81.6) | 198 (83.2) | 520 (82.3) | 1290 (81.1) | 653 (81.4) | 204 (82.6) | 0.91 | 3509 |
| Tolvaptan* | 377 (10.7) | 17 (7.1) | 67 (10.6) | 188 (11.8) | 75 (9.4) | 30 (12.2) | 0.12 | 3509 |
| **Congestion at discharge** |  |  |  |  |  |  |  |  |
| Edema | 419 (12.3) | 28 (12.3) | 55 (9.1) | 197 (12.8) | 85 (11.0) | 54 (22.2) | <0.001 | 3399 |
| Pulmonary congestion | 270 (7.8) | 23 (9.8) | 50 (8.1) | 131 (8.3) | 39 (5.0) | 27 (11.0) | 0.006 | 3457 |
| Jugular venous distention | 224 (6.6) | 15 (6.6) | 37 (6.1) | 115 (7.5) | 34 (4.4) | 23 (9.5) | 0.02 | 3377 |

Values are number (%), mean ± SD, or median (interquartile range). P values were calculated using the chi square test or Fisher’s exact test for categorical variables, and 1-way ANOVA or Kruskal-Wallis test for continuous variables.

* Risk-adjusting variables selected for the Cox proportional hazard models.

Chronic kidney disease was defined as estimated glomerular filtration rate (eGFR) <60 mL/min/1.73 m^2^. Renal dysfunction was defined as estimated glomerular filtration rate (eGFR) <30 mL/min/1.73 m^2^ based on the chronic kidney disease grades.

Anemia was defined using the World Health Organization criteria (hemoglobin <12.0 g/dl in women and <13.0 g/dl in men).

Abbreviations: ACEI, angiotensin-converting enzyme inhibitor; ACS, acute coronary syndrome; ARB, angiotensin-receptor blocker; BNP, brain-type natriuretic peptide; BMI, body mass index; BP, blood pressure; eGFR, estimated glomerular filtration rate; HFmrEF, heart failure with mid-range ejection fraction; HFpEF, heart failure with preserved ejection fraction; HFrEF, heart failure with reduced ejection fraction; LVEF, left ventricular ejection fraction; NT-pro BNP; N-terminal-pro brain-type natriuretic peptide; NYHA, New York Heart Association

**Supplementary Table 4. Baseline characteristics of the study subjects and transthoracic echocardiography results of the patients classified into BMI quartiles at discharge**

| **Variables** | **Total**  **(N=3509)** | **BMI<18.5 (N=870)** | **18.5≤BMI<20.9 (N=889)** | **20.9≤BMI<23.6 (N=863)** | **23.6≤BMI (N=887)** | **P value** | **Total N** |
| --- | --- | --- | --- | --- | --- | --- | --- |
| **Clinical Characteristic** |  |  |  |  |  |  |  |
| Age*, years | 77.2±12.0 | 80.8±10.5 | 79.1±10.8 | 76.8±11.2 | 72.3±13.6 | <0.001 | 3509 |
| Age ≥80 years | 1770 (50.4) | 553 (63.6) | 507 (57.0) | 393 (45.5) | 317 (35.7) | <0.001 | 3509 |
| Women* | 1538 (43.8) | 488 (56.1) | 384 (43.2) | 319 (37.0) | 347 (39.1) | <0.001 | 3509 |
| Body weight, kg at admission | 56.7±14.6 | 44.2±8.1 | 52.1±8.4 | 58.7±8.8 | 71.5±15.5 | <0.001 | 3481 |
| BMI at admission | 22.9±4.5 | 18.4±2.2 | 21.3±1.6 | 23.6±1.7 | 28.3±4.2 | <0.001 | 3481 |
| Body weight, kg at discharge | 52.8±13.5 | 40.0±6.2 | 48.3±6.7 | 55.2±7.3 | 67.6±13.5 | <0.001 | 3509 |
| BMI at discharge | 21.4±4.2 | 16.7±1.4 | 19.7±0.7 | 22.2±0.8 | 26.9±3.5 | <0.001 | 3509 |
| **Etiology** |  |  |  |  |  | <0.001 | 3509 |
| Ischemic | 1147 (32.7) | 232 (26.7) | 278 (31.3) | 298 (34.5) | 339 (38.2) |  |  |
| Associated with ACS* | 194 (5.5) | 28 (3.2) | 41 (4.6) | 66 (7.7) | 59 (6.7) |  |  |
| Not associated with ACS | 953 (27.2) | 204 (23.5) | 237 (26.7) | 232 (26.9) | 280 (31.6) |  |  |
| Hypertensive | 870 (24.8) | 181 (20.8) | 219 (24.6) | 226 (26.2) | 244 (27.5) |  |  |
| Valvular heart disease | 683 (19.5) | 244 (28.1) | 179 (20.1) | 151 (17.5) | 109 (12.3) |  |  |
| Cardiomyopathy | 534 (15.2) | 136 (15.6) | 137 (15.4) | 125 (14.5) | 136 (15.3) |  |  |
| Dilated cardiomyopathy | 386 (11.0) | 92 (10.6) | 98 (11.0) | 89 (10.3) | 107 (12.1) |  |  |
| Arrhythmia-related | 164 (4.7) | 44 (5.1) | 48 (5.4) | 37 (4.3) | 35 (4.0) |  |  |
| **Medical history** |  |  |  |  |  |  |  |
| Heart failure hospitalization* | 1272 (36.8) | 348 (40.3) | 329 (37.4) | 293 (34.8) | 302 (34.6) | 0.047 | 3456 |
| Hypertension* | 2551 (72.7) | 562 (64.6) | 640 (72.0) | 653 (75.7) | 696 (78.5) | <0.001 | 3509 |
| Diabetes* | 1327 (37.8) | 193 (22.2) | 306 (34.4) | 362 (42.0) | 466 (52.5) | <0.001 | 3509 |
| Dyslipidemia | 1393 (39.7) | 262 (30.1) | 346 (38.9) | 337 (39.1) | 448 (50.5) | <0.001 | 3509 |
| Atrial fibrillation or flutter* | 1477 (42.1) | 376 (43.2) | 379 (42.6) | 353 (40.9) | 369 (41.6) | 077 | 3509 |
| VT/VF | 147 (4.2) | 35 (4.0) | 40 (4.5) | 31 (3.6) | 41 (4.6) | 0.69 | 3509 |
| Previous myocardial infarction* | 806 (23.0) | 172 (19.8) | 203 (22.8) | 200 (23.2) | 231 (26.0) | 0.02 | 3509 |
| Prior PCI or CABG | 924 (26.3) | 183 (21.0) | 235 (26.4) | 226 (26.2) | 280 (31.6) | <0.001 | 3509 |
| Previous stroke* | 548 (15.6) | 138 (15.9) | 142 (16.0) | 142 (16.5) | 126 (14.2) | 0.59 | 3509 |
| Current smoking* | 442 (12.8) | 88 (10.3) | 99 (11.3) | 111 (13.1) | 144 (16.5) | <0.001 | 3450 |
| Chronic lung disease* | 463 (13.2) | 127 (14.6) | 106 (11.9) | 112 (13.0) | 118 (13.3) | 0.42 | 3509 |
| COPD | 289 (8.2) | 97 (11.2) | 74 (8.3) | 64 (7.4) | 54 (6.1) | 0.001 | 3509 |
| Liver cirrhosis | 46 (1.3) | 3 (0.3) | 19 (2.1) | 11 (1.3) | 13 (1.5) | 0.01 | 3509 |
| Malignancy * | 507 (14.4) | 154 (17.7) | 129 (14.5) | 125 (14.5) | 99 (11.2) | 0.002 | 3509 |
| Dementia | 569 (16.2) | 200 (23.0) | 153 (17.2) | 133 (15.4) | 83 (9.4) | <0.001 | 3509 |
| **Social background on admission** |  |  |  |  |  |  |  |
| Poor medical adherence | 586 (16.7) | 148 (17.0) | 139 (15.6) | 139 (16.1) | 160 (18.0) | 0.54 | 3509 |
| Living alone* | 755 (21.5) | 190 (21.8) | 184 (20.7) | 169 (19.6) | 212 (23.9) | 0.15 | 3509 |
| Employed | 484 (13.8) | 71 (8.2) | 80 (9.0) | 134 (15.5) | 199 (22.4) | <0.001 | 3509 |
| Public financial assistance | 207 (5.9) | 52 (6.0) | 52 (5.9) | 47 (5.5) | 56 (6.3) | 0.89 | 3509 |
| **Daily life activities** |  |  |  |  |  | <0.001 | 3475 |
| Ambulatory* | 2837 (81.6) | 636 (74.0) | 714 (81.4) | 732 (85.5) | 755 (85.5) |  |  |
| Use of wheelchair (outdoor only) | 251 (7.2) | 69 (8.0) | 72 (8.2) | 59 (6.9) | 51 (5.8) |  |  |
| Use of wheelchair (outdoor and indoor) | 297 (8.5) | 117 (13.6) | 72 (8.2) | 53 (6.2) | 55 (6.2) |  |  |
| Bedridden | 90 (2.6) | 37 (4.3) | 19 (2.2) | 12 (1.4) | 22 (2.5) |  |  |
| **Vital signs at presentation** |  |  |  |  |  |  |  |
| Heart rate, bpm | 96.0±27.6 | 95.4±26.7 | 94.9±27.9 | 96.7±26.8 | 96.8±28.9 | 0.48 | 3488 |
| <60 beats/min* | 232 (6.7) | 58 (6.7) | 66 (7.5) | 48 (5.6) | 60 (6.8) | 0.47 | 3488 |
| Systolic BP, mmHg | 148.3±34.9 | 144.0±33.7 | 146.9±34.4 | 149.9±35.3 | 152.2±35.7 | <0.001 | 3500 |
| Systolic BP <90 mm Hg* | 87 (2.5) | 28 (3.2) | 28 (3.2) | 15 (1.7) | 16 (1.8) | 0.06 | 3500 |
| Diastolic BP, mmHg | 85.3±23.8 | 82.7±23.4 | 83.4±23.4 | 86.7±23.4 | 88.2±24.8 | <0.001 | 3494 |
| **Rhythms at presentation** |  |  |  |  |  | 0.09 | 3509 |
| Sinus Rhythm | 1964 (56.0) | 500 (57.5) | 489 (55.0) | 468 (54.2) | 507 (57.2) |  |  |
| Atrial fibrillation or flutter | 1271 (36.2) | 311 (35.8) | 311 (35.0) | 327 (37.9) | 322 (36.3) |  |  |
| NYHA class III or IV | 3042 (86.9) | 772 (88.9) | 758 (85.5) | 730 (84.9) | 782 (88.5) | 0.02 | 3499 |
| **Test results at admission** |  |  |  |  |  |  |  |
| LVEF, % | 46.2±16.2 | 45.6±16.4 | 46.1±16.5 | 46.2±15.7 | 47.0±16.3 | 0.41 | 3435 |
| LVEF classification |  |  |  |  |  | 0.90 | 3498 |
| HFrEF (LVEF<40%)* | 1321 (37.8) | 333 (38.3) | 339 (38.3) | 325 (37.8) | 324 (36.7) |  |  |
| HFmrEF (LVEF40%-49%) | 659 (18.8) | 163 (18.8) | 165 (18.6) | 171 (19.9) | 160 (18.1) |  |  |
| HFpEF (LVEF≥50%) | 1518 (43.4) | 373 (42.9) | 382 (43.1) | 363 (42.3) | 400 (45.3) |  |  |
| BNP, pg/ml | 710 (387-1251) | 933 (511-1628) | 778 (452-1436) | 679 (389-1170) | 498 (283-876) | <0.001 | 3108 |
| NT-proBNP, pg/ml | 5416 (2631-11955) | 9663 (4192-17340) | 6721 (3505-11796) | 4036 (2375-8777) | 4012 (1935-7683) | <0.001 | 614 |
| Serum creatinine, mg/dl | 1.48±1.28 | 1.31±0.99 | 1.53±1.32 | 1.56±1.41 | 1.51±1.32 | <0.001 | 3503 |
| eGFR, ml/min/1.73m^2^ | 46.3±23.4 | 48.2±24.9 | 44.6±23.0 | 45.6±22.4 | 46.7±23.0 | 0.03 | 3503 |
| <60 ml/min/1.73m^2^ | 2588 (73.9) | 618 (71.3) | 685 (77.1) | 641 (74.4) | 644 (72.7) | 0.03 | 3503 |
| <30 ml/min/1.73m^2^* | 921 (26.3) | 224 (25.8) | 254 (28.6) | 217 (25.2) | 226 (25.5) | 0.34 | 3503 |
| Blood urea nitrogen, mg/dl | 28.3±16.1 | 29.3±15.9 | 28.9±15.9 | 28.8±17.2 | 26.2±15.2 | <0.001 | 3498 |
| Albumin, g/dl | 3.49±0.49 | 3.36±0.46 | 3.48±0.48 | 3.54±0.48 | 3.60±0.50 | <0.001 | 3408 |
| <3.0 g/dl* | 438 (12.9) | 148 (17.5) | 117 (13.6) | 94 (11.2) | 79 (9.2) | <0.001 | 3408 |
| Sodium, mEq/l | 139.2±4.1 | 138.8±4.6 | 139.1±4.1 | 139.2±4.0 | 139.5±3.8 | 0.009 | 3498 |
| <135 mEq/l* | 405 (11.6) | 134 (15.5) | 111 (12.5) | 80 (9.3) | 80 (9.1) | <0.001 | 3498 |
| Hemoglobin, g/dl | 11.6±2.4 | 11.0±2.1 | 11.4±2.3 | 11.8±2.3 | 12.2±2.5 | <0.001 | 3503 |
| Anemia* | 2299 (65.6) | 650 (74.8) | 617 (69.6) | 540 (62.7) | 492 (55.5) | <0.001 | 3503 |
| CRP, mg/dL | 1.99±3.58 | 2.12±3.53 | 1.96±3.47 | 2.07±3.97 | 1.80±3.32 | 0.50 | 3422 |
| **Medication at discharge** |  |  |  |  |  |  |  |
| ACEI or ARB* | 2058 (58.6) | 438 (50.3) | 504 (56.7) | 523 (60.6) | 593 (66.9) | <0.001 | 3509 |
| β blocker* | 2376 (67.7) | 525 (60.3) | 596 (67.0) | 594 (68.8) | 661 (74.5) | <0.001 | 3509 |
| MRA | 1589 (45.3) | 428 (49.2) | 373 (42.0) | 379 (43.9) | 409 (46.1) | 0.02 | 3509 |
| Loop diuretics | 2865 (81.6) | 718 (82.5) | 712 (80.1) | 715 (82.9) | 720 (81.2) | 0.41 | 3509 |
| Tolvaptan* | 377 (10.7) | 84 (9.7) | 109 (12.3) | 93 (10.8) | 91 (10.3) | 0.33 | 3509 |
| **Congestion at discharge** |  |  |  |  |  |  |  |
| Edema | 419 (12.3) | 83 (9.9) | 107 (12.4) | 108 (12.9) | 121 (14.1) | 0.07 | 3399 |
| Pulmonary congestion | 270 (7.8) | 73 (8.6) | 82 (9.4) | 61 (7.2) | 54 (6.2) | 0.06 | 3457 |
| Jugular venous distention | 224 (6.6) | 52 (6.3) | 73 (8.5) | 48 (5.7) | 51 (6.0) | 0.08 | 3377 |

Values are number (%), mean ± SD, or median (interquartile range). P values were calculated using the chi square test or Fisher’s exact test for categorical variables, and 1-way ANOVA or Kruskal-Wallis test for continuous variables.

* Risk-adjusting variables selected for the Cox proportional hazard models.

Chronic kidney disease was defined as estimated glomerular filtration rate (eGFR) <60 mL/min/1.73 m^2^. Renal dysfunction was defined as estimated glomerular filtration rate (eGFR) <30 mL/min/1.73 m^2^ based on the chronic kidney disease grades.

Anemia was defined using the World Health Organization criteria (hemoglobin <12.0 g/dl in women and <13.0 g/dl in men).

Abbreviations: ACEI, angiotensin-converting enzyme inhibitor; ACS, acute coronary syndrome; ARB, angiotensin-receptor blocker; BNP, brain-type natriuretic peptide; BMI, body mass index; BP, blood pressure; eGFR, estimated glomerular filtration rate; HFmrEF, heart failure with mid-range ejection fraction; HFpEF, heart failure with preserved ejection fraction; HFrEF, heart failure with reduced ejection fraction; LVEF, left ventricular ejection fraction; NT-pro BNP; N-terminal-pro brain-type natriuretic peptide; NYHA, New York Heart Association

**Supplementary Table 5. Baseline characteristics of the study subjects and transthoracic echocardiography results of the patients in BMI classification at admission**

| **Variables** | **Total**  **(N=3828)** | **Severely underweight (N=117)** | **Underweight (N=432)** | **Normal weight**  **(N=2287)** | **Overweight (N=760)** | **Obese**  **(N=232)** | **P value** | **Total N** |
| --- | --- | --- | --- | --- | --- | --- | --- | --- |
| **Clinical Characteristic** |  |  |  |  |  |  |  |  |
| Age*, years | 77.6±12.1 | 82.9±10.8 | 81.7±10.3 | 78.7±10.8 | 74.3±12.5 | 66.8±16.3 | <0.001 | 3828 |
| Age ≥80 years | 1984 (51.8) | 89 (76.1) | 293 (67.8) | 1241 (54.3) | 300 (39.5) | 61 (26.3) | <0.001 | 3828 |
| Women* | 1685 (44.0) | 74 (63.3) | 257 (59.5) | 1003 (43.9) | 255 (33.6) | 96 (41.4) | <0.001 | 3828 |
| Body weight, kg at admission | 56.3±14.6 | 35.6±4.6 | 41.5±5.8 | 53.2±8.6 | 68.0±9.8 | 86.7±17.5 | <0.001 | 3828 |
| BMI at admission | 22.8±4.5 | 14.9±0.9 | 17.4±0.7 | 21.8±1.8 | 26.9±1.3 | 34.0±4.2 | <0.001 | 3828 |
| Body weight, kg at discharge | 52.8±13.5 | 34.6±4.7 | 39.8±6.6 | 49.9±8.6 | 62.5±9.7 | 79.5±16.7 | <0.001 | 3543 |
| BMI at discharge | 21.4±4.2 | 14.6±1.2 | 16.7±1.7 | 20.4±2.1 | 24.8±2.1 | 31.0±4.4 | <0.001 | 3543 |
| **Etiology** |  |  |  |  |  |  | <0.001 | 3828 |
| Ischemic | 1255 (32.8) | 30 (25.6) | 109 (25.2) | 754 (33.0) | 286 (37.6) | 76 (32.8) |  |  |
| Associated with ACS* | 217 (5.7) | 7 (6.0) | 11 (2.6) | 133 (5.8) | 52 (6.8) | 14 (6.0) |  |  |
| Not associated with ACS | 1038 (27.1) | 23 (19.7) | 98 (22.7) | 621 (27.2) | 234 (30.8) | 62 (26.7) |  |  |
| Hypertensive | 924 (24.1) | 25 (21.4) | 92 (21.3) | 530 (23.2) | 206 (27.1) | 71 (30.6) |  |  |
| Valvular heart disease | 765 (20.0) | 35 (29.9) | 119 (27.6) | 477 (20.9) | 106 (14.0) | 28 (12.1) |  |  |
| Cardiomyopathy | 582 (15.2) | 18 (15.4) | 72 (16.7) | 342 (15.0) | 109 (14.3) | 41 (17.7) |  |  |
| Dilated cardiomyopathy | 417 (10.9) | 11 (9.4) | 48 (11.1) | 237 (10.4) | 88 (11.6) | 33 (14.2) |  |  |
| Arrhythmia-related | 177 (4.6) | 5 (4.3) | 19 (4.4) | 113 (4.9) | 33 (4.3) | 7 (3.0) |  |  |
| **Medical history** |  |  |  |  |  |  |  |  |
| Heart failure hospitalization* | 1393 (37.0) | 47 (40.2) | 168 (39.5) | 839 (37.5) | 257 (34.2) | 82 (36.0) | 0.35 | 3761 |
| Hypertension* | 2750 (71.8) | 69 (59.0) | 289 (66.9) | 1640 (71.7) | 574 (75.5) | 178 (76.7) | <0.001 | 3828 |
| Diabetes* | 1432 (37.4) | 19 (16.2) | 84 (19.4) | 811 (35.5) | 382 (50.3) | 136 (58.6) | <0.001 | 3828 |
| Dyslipidemia | 1478 (38.6) | 28 (23.9) | 130 (30.1) | 843 (36.9) | 357 (47.0) | 120 (51.7) | <0.001 | 3828 |
| Atrial fibrillation or flutter* | 1600 (41.8) | 45 (38.5) | 178 (41.2) | 956 (41.8) | 328 (43.2) | 93 (40.1) | 0.83 | 3828 |
| VT/VF | 160 (4.2) | 3 (2.6) | 20 (4.6) | 92 (4.0) | 32 (4.2) | 13 (5.6) | 0.68 | 3828 |
| Previous myocardial infarction* | 870 (22.7) | 21 (18.0) | 83 (19.2) | 523 (22.9) | 194 (25.5) | 49 (21.1) | 0.08 | 3828 |
| Prior PCI or CABG | 984 (25.7) | 22 (18.8) | 86 (19.9) | 586 (25.6) | 222 (29.2) | 68 (29.3) | 0.002 | 3828 |
| Previous stroke* | 611 (16.0) | 13 (11.1) | 75 (17.4) | 364 (15.9) | 129 (17.0) | 30 (12.9) | 0.30 | 3828 |
| Current smoking* | 466 (12.4) | 16 (13.9) | 45 (10.6) | 251 (11.2) | 115 (15.4) | 39 (16.8) | 0.005 | 3760 |
| Chronic lung disease* | 511 (13.3) | 18 (15.4) | 75 (17.4) | 285 (12.5) | 101 (13.3) | 32 (13.8) | 0.09 | 3828 |
| COPD | 318 (8.3) | 16 (13.7) | 62 (14.4) | 180 (7.9) | 52 (6.8) | 8 (3.5) | <0.001 | 3828 |
| Liver cirrhosis | 53 (1.4) | 0 (0) | 4 (0.9) | 34 (1.5) | 11 (1.5) | 4 (1.7) | 0.61 | 3828 |
| Malignancy * | 553 (14.4) | 27 (23.1) | 61 (14.1) | 338 (14.8) | 108 (14.2) | 19 (8.2) | 0.005 | 3828 |
| Dementia | 681 (17.8) | 39 (33.3) | 124 (28.7) | 412 (18.0) | 92 (12.1) | 14 (6.0) | <0.001 | 3828 |
| **Social background on admission** |  |  |  |  |  |  |  |  |
| Poor medical adherence | 634 (16.6) | 17 (14.5) | 71 (16.4) | 368 (16.1) | 141 (18.6) | 37 (16.0) | 0.56 | 3828 |
| Living alone* | 824 (21.5) | 21 (18.0) | 93 (21.5) | 476 (20.8) | 175 (23.0) | 59 (25.4) | 0.32 | 3828 |
| Employed | 497 (13.0) | 4 (3.4) | 30 (6.9) | 257 (11.2) | 136 (17.9) | 70 (30.2) | <0.001 | 3828 |
| Public financial assistance | 227 (5.9) | 5 (4.3) | 28 (6.5) | 127 (5.6) | 49 (6.5) | 18 (7.8) | 0.53 | 3828 |
| **Daily life activities** |  |  |  |  |  |  | <0.001 | 3794 |
| Ambulatory* | 3035 (80.0) | 74 (63.3) | 288 (67.9) | 1843 (81.4) | 632 (83.5) | 198 (85.7) |  |  |
| Use of wheelchair (outdoor only) | 278 (7.3) | 6 (5.1) | 37 (8.7) | 166 (7.3) | 54 (7.1) | 15 (6.5) |  |  |
| Use of wheelchair (outdoor and indoor) | 349 (9.2) | 26 (22.2) | 69 (16.3) | 191 (8.4) | 55 (7.3) | 8 (3.5) |  |  |
| Bedridden | 132 (3.5) | 11 (9.4) | 30 (7.1) | 65 (2.9) | 16 (2.1) | 10 (4.3) |  |  |
| **Vital signs at presentation** |  |  |  |  |  |  |  |  |
| Heart rate, bpm | 95.9±27.5 | 96.8±23.4 | 94.4±26.0 | 96.3±27.8 | 95.5±28.3 | 96.6±27.1 | 0.54 | 3801 |
| <60 beats/min* | 248 (6.5) | 7 (6.0) | 31 (7.2) | 138 (6.1) | 56 (7.4) | 16 (6.9) | 0.71 | 3801 |
| Systolic BP, mmHg | 147.1±35.1 | 139.7±35.0 | 146.4±36.3 | 146.6±35.0 | 149.6±34.8 | 149.2±34.6 | 0.03 | 3817 |
| Systolic BP <90 mm Hg* | 113 (3.0) | 5 (4.3) | 15 (3.5) | 72 (3.2) | 18 (2.4) | 3 (1.3) | 0.33 | 3819 |
| Diastolic BP, mmHg | 84.7±23.9 | 78.2±22.1 | 83.9±25.5 | 84.2±23.4 | 86.3±24.1 | 88.1±25.1 | 0.001 | 3810 |
| **Rhythms at presentation** |  |  |  |  |  |  | 0.12 | 3828 |
| Sinus Rhythm | 2142 (56.0) | 78 (66.7) | 255 (59.0) | 1256 (54.9) | 418 (55.0) | 135 (58.2) |  |  |
| Atrial fibrillation or flutter | 1382 (36.1) | 35 (29.9) | 140 (32.4) | 833 (36.4) | 291 (38.3) | 83 (35.8) |  |  |
| NYHA class III or IV | 3336 (87.4) | 108 (92.3) | 378 (88.1) | 1981 (86.9) | 664 (87.5) | 205 (89.5) | 0.36 | 3815 |
| **Test results at admission** |  |  |  |  |  |  |  |  |
| LVEF, % | 45.9±16.3 | 44.3±16.7 | 44.7±16.8 | 46.0±16.1 | 46.9±16.4 | 45.7±16.8 | 0.19 | 3712 |
| LVEF classification |  |  |  |  |  |  | 0.70 | 3814 |
| HFrEF (LVEF<40%)* | 1476 (38.7) | 47 (40.2) | 181 (42.0) | 874 (38.4) | 280 (36.9) | 94 (40.9) |  |  |
| HFmrEF (LVEF40%-49%) | 703 (18.4) | 20 (17.1) | 77 (17.9) | 432 (19.0) | 13 (18.3) | 35 (15.2) |  |  |
| HFpEF (LVEF≥50%) | 1635 (42.9) | 50 (42.7) | 173 (40.1) | 972 (42.7) | 339 (44.7) | 101 (43.9) |  |  |
| BNP, pg/ml | 717 (393-1294) | 1176 (585-1904) | 1025 (521-1700) | 751 (435-1344) | 522 (290-923) | 461 (242-796) | <0.001 | 3387 |
| NT-proBNP, pg/ml | 5702 (2665-12434) | 9998 (3216-22443) | 10856 (5979-18098) | 5784 (2920-11880) | 4622 (2020-9000) | 2636 (1302-5465) | <0.001 | 656 |
| Serum creatinine, mg/dl | 1.49±1.27 | 1.30±0.87 | 1.35±1.06 | 1.53±1.37 | 1.48±1.19 | 1.46±0.99 | 0.01 | 3821 |
| eGFR, ml/min/1.73m^2^ | 45.8±23.4 | 46.8±25.3 | 46.8±25.0 | 45.2±23.1 | 46.7±23.5 | 46.9±23.0 | 0.56 | 3821 |
| <60 ml/min/1.73m^2^ | 2853 (74.7) | 89 (76.1) | 309 (72.2) | 1727 (75.6) | 554 (73.0) | 174 (75.0) | 0.46 | 3821 |
| <30 ml/min/1.73m^2^* | 1037 (27.1) | 32 (27.4) | 117 (27.3) | 629 (27.5) | 202 (26.6) | 57 (24.6) | 0.90 | 3821 |
| Blood urea nitrogen, mg/dl | 29.0±16.9 | 33.7±19.7 | 30.6±16.0 | 29.3±17.0 | 27.1±16.4 | 27.0±16.6 | <0.001 | 3817 |
| Albumin, g/dl | 3.47±0.50 | 3.26±0.54 | 3.35±0.49 | 3.48±0.48 | 3.53±0.52 | 3.53±0.53 | <0.001 | 3714 |
| <3.0 g/dl* | 524 (14.1) | 27 (23.7) | 79 (18.9) | 293 (13.2) | 93 (12.5) | 32 (14.2) | <0.001 | 3714 |
| Sodium, mEq/l | 139.1±4.3 | 139.4±4.7 | 138.2±4.6 | 139.1±4.2 | 139.3±4.3 | 139.2±4.2 | <0.001 | 3817 |
| <135 mEq/l* | 479 (12.5) | 14 (12.0) | 82 (19.1) | 280 (12.3) | 77 (10.1) | 26 (11.3) | <0.001 | 3817 |
| Hemoglobin, g/dl | 11.6±2.4 | 10.7±2.1 | 11.0±2.1 | 11.5±2.3 | 12.0±2.5 | 12.5±2.7 | <0.001 | 3821 |
| Anemia* | 2540 (66.5) | 90 (76.9) | 324 (75.0) | 1550 (67.9) | 455 (60.0) | 121 (52.2) | <0.001 | 3821 |
| CRP, mg/dL | 2.07±3.66 | 2.95±4.45 | 2.20±3.47 | 2.02±3.59 | 2.05±3.89 | 2.01±3.33 | 0.02 | 3738 |
| **Medication at admission** |  |  |  |  |  |  |  |  |
| ACEI or ARB* | 1758 (45.9) | 36 (30.8) | 166 (38.4) | 1022 (44.7) | 407 (53.6) | 127 (54.7) | <0.001 | 3828 |
| β blocker* | 1495 (39.1) | 34 (29.1) | 137 (31.7) | 904 (39.5) | 318 (41.8) | 102 (44.0) | <0.001 | 3828 |
| MRA | 696 (18.2) | 24 (20.5) | 99 (22.9) | 403 (17.6) | 131 (17.2) | 39 (16.8) | 0.09 | 3828 |
| Loop diuretics | 1881 (49.1) | 53 (45.3) | 211 (48.8) | 1107 (48.4) | 386 (50.8) | 124 (53.5) | 0.44 | 3828 |
| Tolvaptan* | 163 (4.3) | 4 (3.4) | 15 (3.5) | 104 (4.6) | 29 (3.8) | 11 (4.7) | 0.77 | 3828 |
| **Congestion at admission** |  |  |  |  |  |  |  |  |
| Edema | 2868 (77.1) | 77 (67.5) | 294 (69.7) | 1688 (76.2) | 609 (82.2) | 200 (88.1) | <0.001 | 3720 |
| Pulmonary congestion | 3515 (92.8) | 103 (88.8) | 394 (92.1) | 2106 (93.0) | 698 (93.2) | 214 (92.6) | 0.49 | 3789 |
| Jugular venous distention | 2814 (78.1) | 92 (84.4) | 329 (79.9) | 1651 (77.0) | 560 (78.1) | 182 (82.4) | 0.13 | 3602 |

Values are number (%), mean ± SD, or median (interquartile range). P values were calculated using the chi square test or Fisher’s exact test for categorical variables, and 1-way ANOVA or Kruskal-Wallis test for continuous variables.

* Risk-adjusting variables selected for the Cox proportional hazard models.

Chronic kidney disease was defined as estimated glomerular filtration rate (eGFR) <60 mL/min/1.73 m^2^. Renal dysfunction was defined as estimated glomerular filtration rate (eGFR) <30 mL/min/1.73 m^2^ based on the chronic kidney disease grades.

Anemia was defined using the World Health Organization criteria (hemoglobin <12.0 g/dl in women and <13.0 g/dl in men).

Abbreviations: ACEI, angiotensin-converting enzyme inhibitor; ACS, acute coronary syndrome; ARB, angiotensin-receptor blocker; BNP, brain-type natriuretic peptide; BMI, body mass index; BP, blood pressure; eGFR, estimated glomerular filtration rate; HFmrEF, heart failure with mid-range ejection fraction; HFpEF, heart failure with preserved ejection fraction; HFrEF, heart failure with reduced ejection fraction; LVEF, left ventricular ejection fraction; NT-pro BNP; N-terminal-pro brain-type natriuretic peptide; NYHA, New York Heart Association

**Supplementary Figure 1. Histograms of BMI value**

BMI, body mass index

**Supplementary Figure 2. Kaplan-Meier curves for the primary and secondary outcome measures using modified classification for Asian populations**

(A) All-cause death (B) Cardiovascular death (C) Non-cardiovascular death (D) Heart failure hospitalization

HF, heart failure

**Supplementary Figure 3. Forrest plots for the adjusted hazard ratios of each BMI category for the clinical outcome measures using modified classification for Asian populations**

BMI, body mass index; CI, confidence interval; HF, heart failure; HR, hazard ratio.

**Supplementary Figure 4. Kaplan-Meier curves for the primary and secondary outcome measures using BMI quartiles at discharge**

(A) All-cause death (B) Cardiovascular death (C) Non-cardiovascular death (D) Heart failure hospitalization

HF, heart failure

**Supplementary Figure 5. Forrest plots for the adjusted hazard ratios of each BMI category for the clinical outcome measures using BMI quartiles at discharge**

BMI, body mass index; CI, confidence interval; HF, heart failure; HR, hazard ratio.

**Supplementary Figure 6. Flowchart regarding BMI at admission**

ADHF, acute decompensated heart failure; BMI, body mass index; KCHF, Kyoto Congestive Heart Failure.

**Supplementary Figure 7. Kaplan-Meier curves for the primary and secondary outcome measures using BMI classification at admission**

(A) All-cause death (B) Cardiovascular death (C) Non-cardiovascular death

**Supplementary Figure 8. Forrest plots for the adjusted hazard ratios of each BMI category for the clinical outcome measures using BMI classification at admission**

BMI, body mass index; CI, confidence interval; HR, hazard ratio.

**Supplementary Figure 1.**


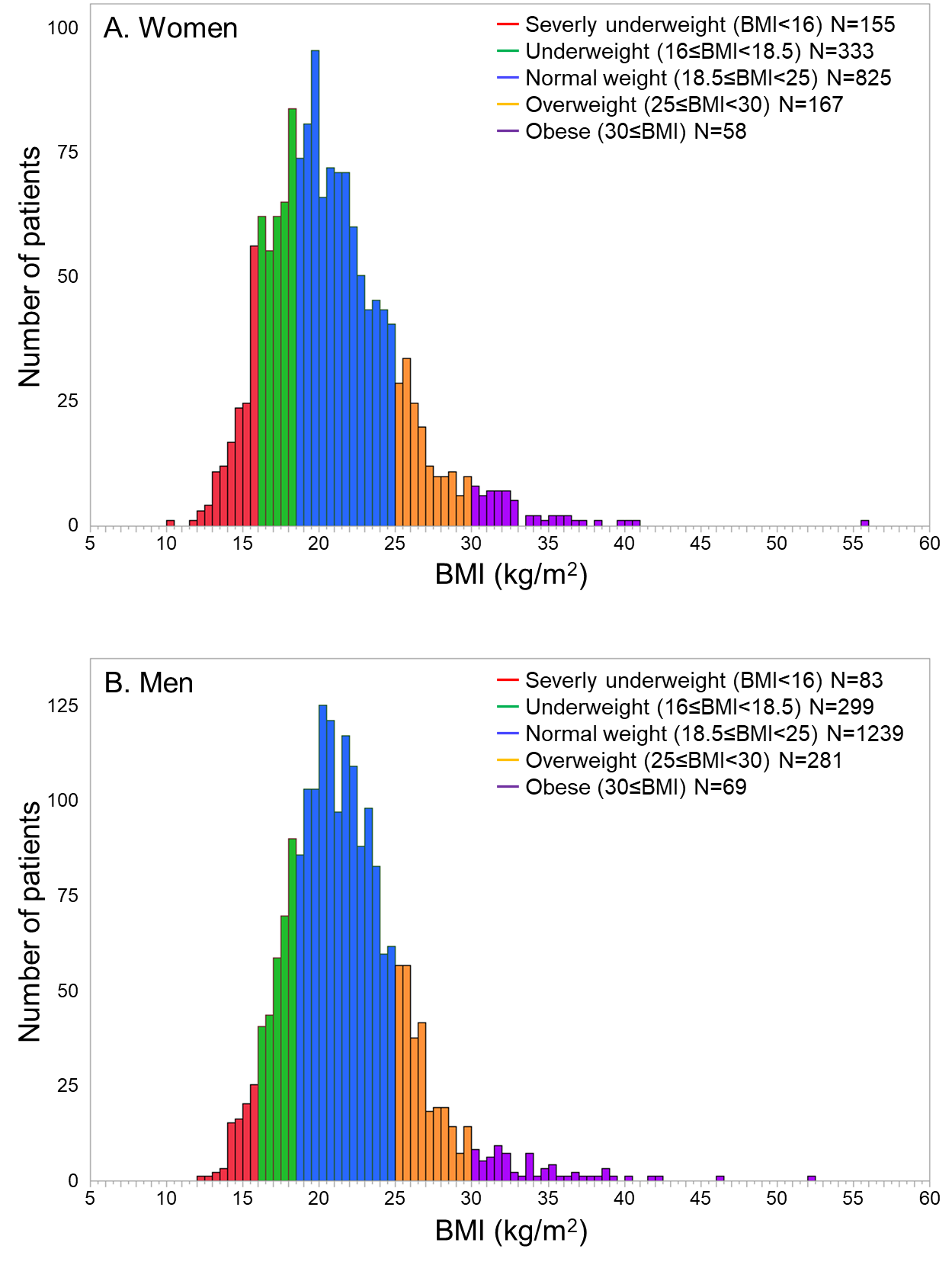


**Supplementary Figure 2.**


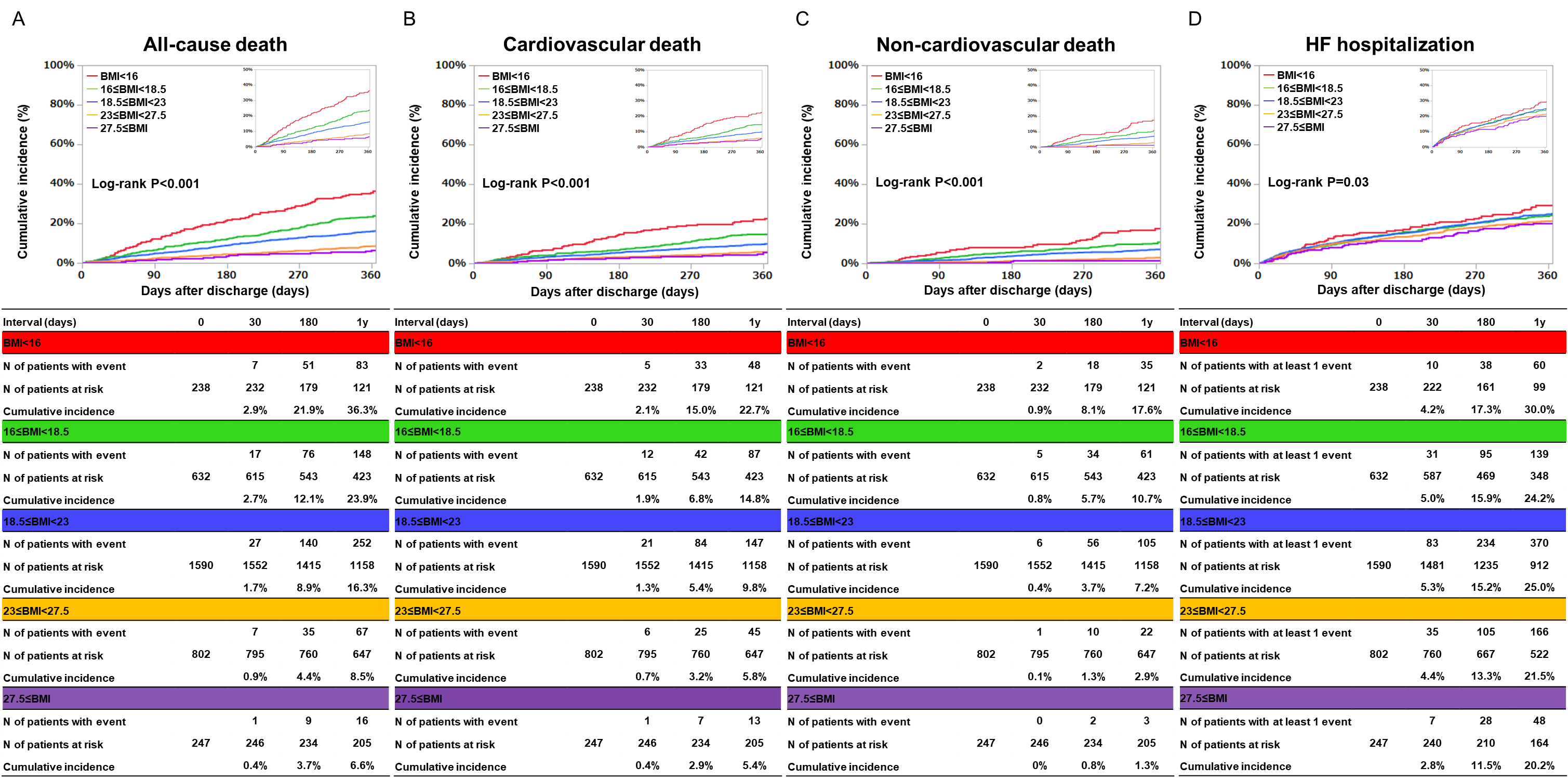


**Supplementary Figure 3.**


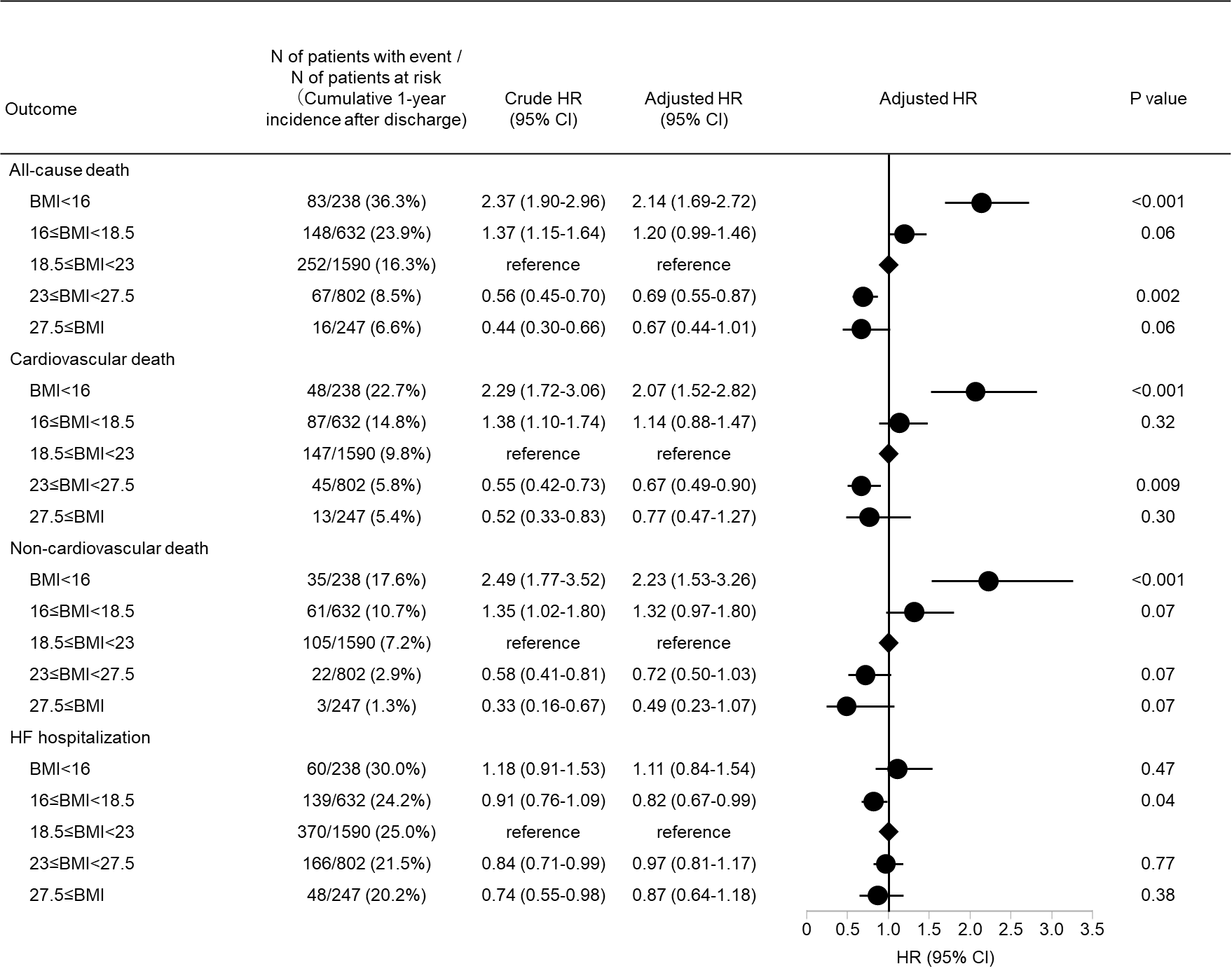


**Supplementary Figure 4.**


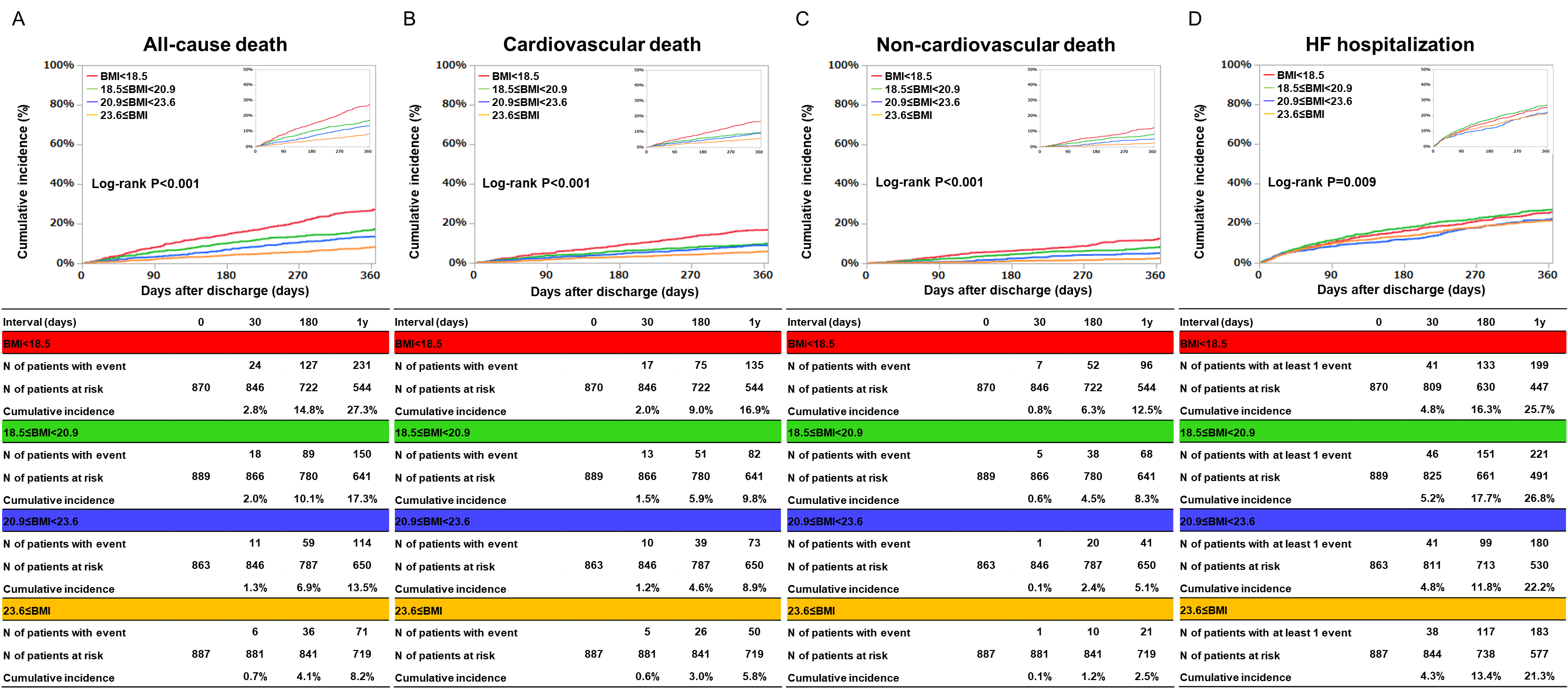


**Supplementary Figure 5.**


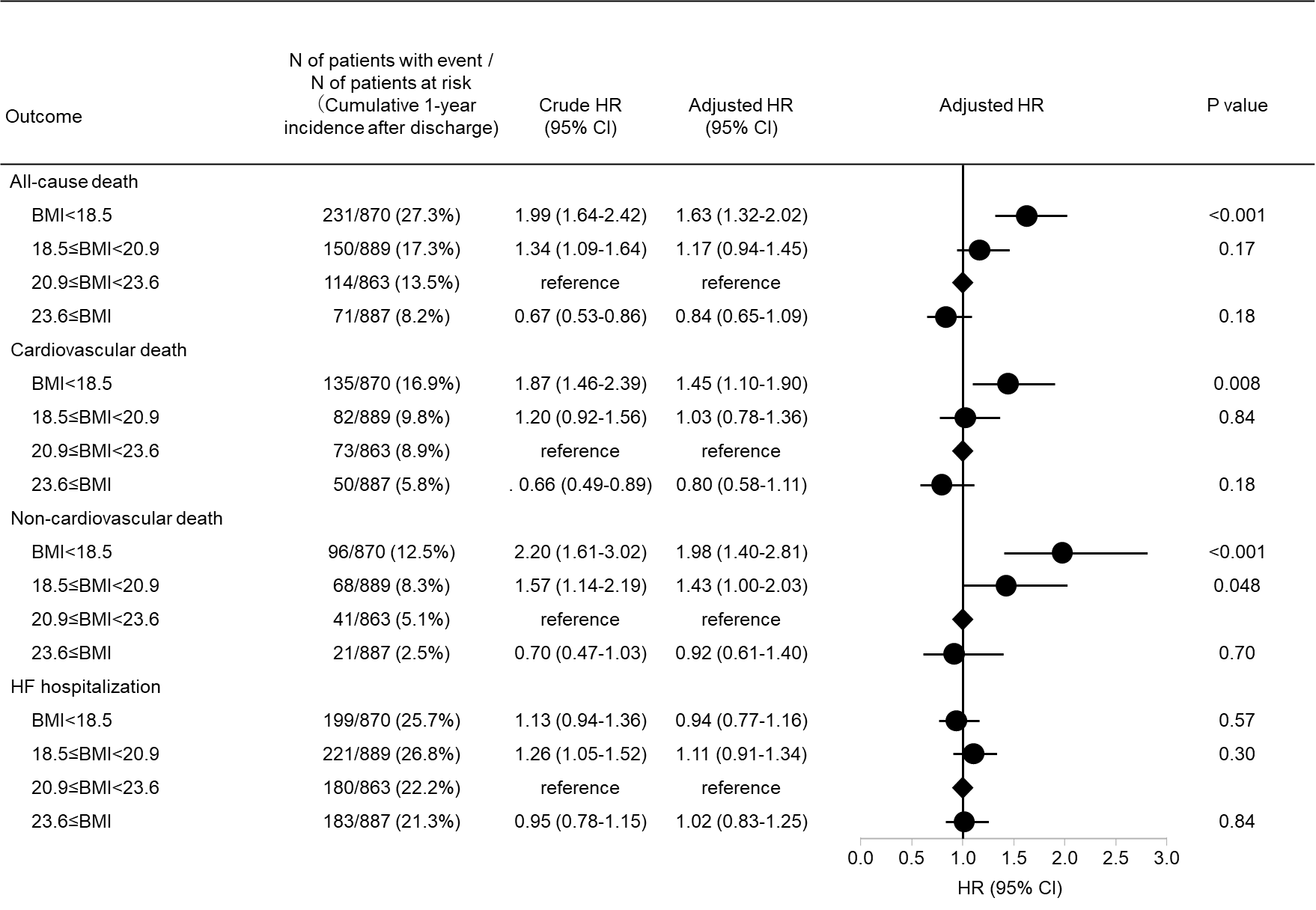


**Supplementary Figure 6.**


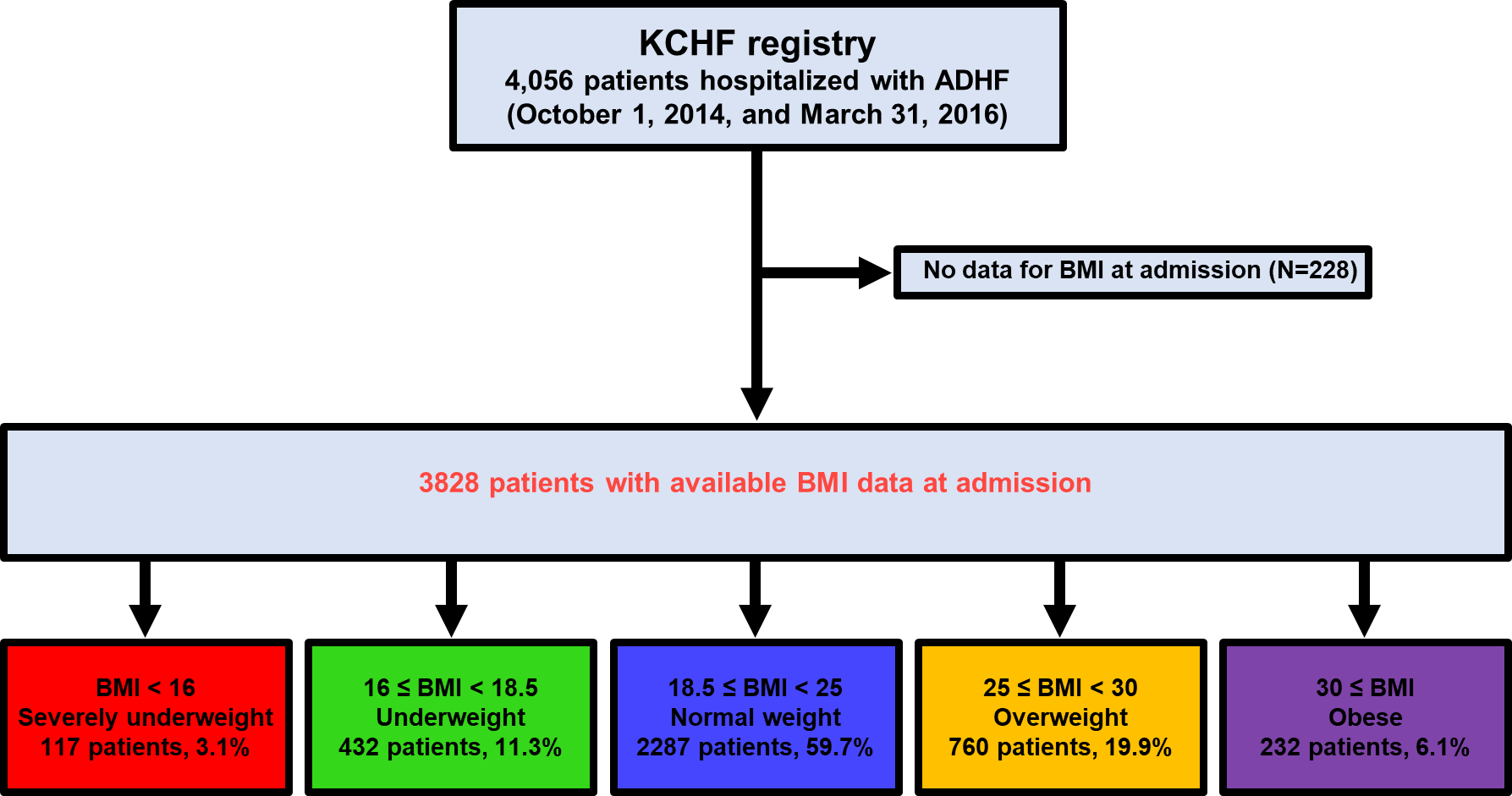


**Supplementary Figure 7.**


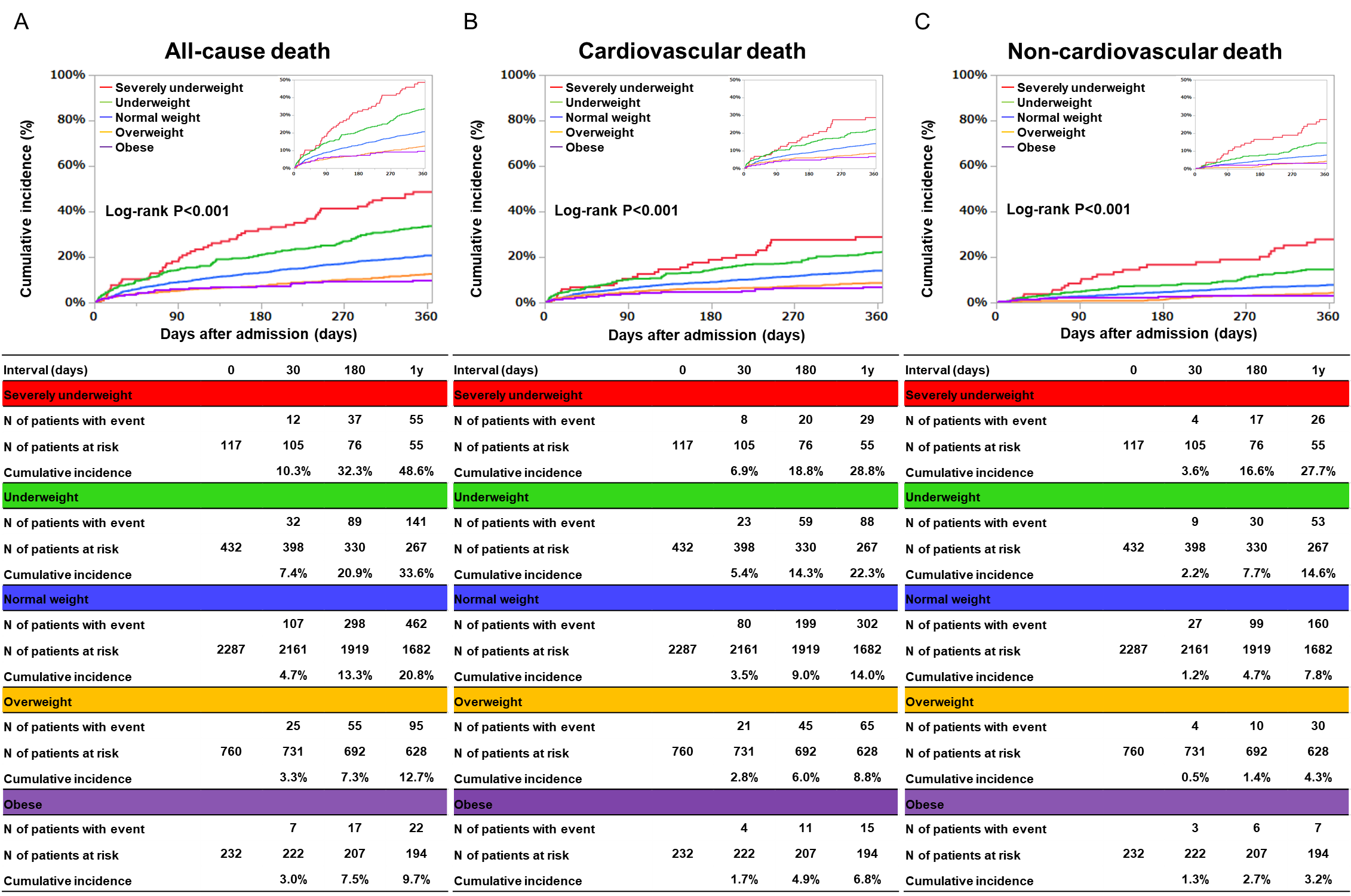


**Supplementary Figure 8.**


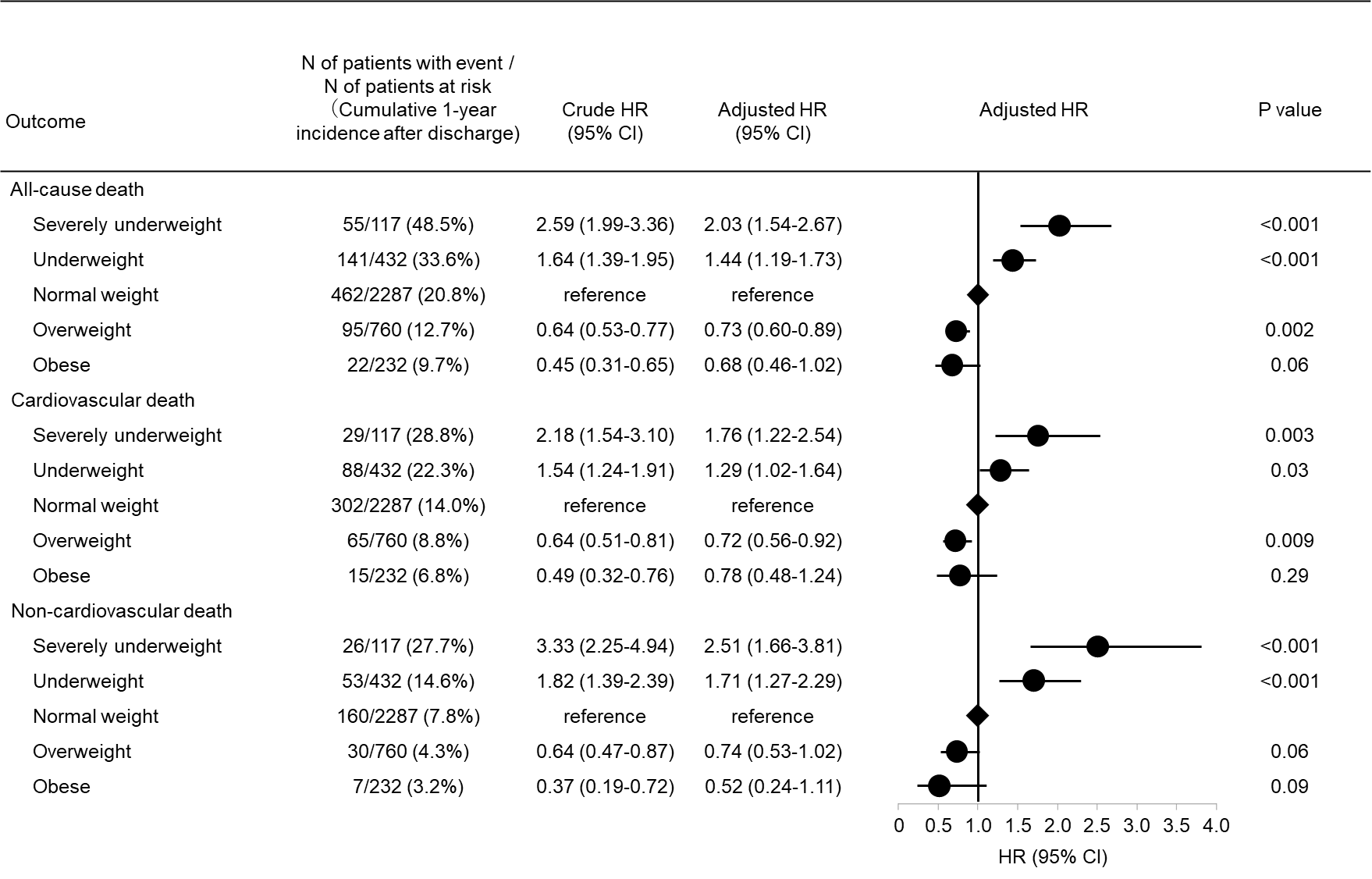

Supplement: Supplementary file 1 — Supplementary Information. [file 41598_2020_73640_MOESM1_ESM.docx]
